# cAMP-independent Crp homolog adds to the multi-layer regulatory network in *Porphyromonas gingivalis*

**DOI:** 10.3389/fcimb.2025.1535009

**Published:** 2025-04-16

**Authors:** Michał Śmiga, Ewa Roszkiewicz, Paulina Ślęzak, Michał Tracz, Teresa Olczak

**Affiliations:** ^1^ Laboratory of Medical Biology, Faculty of Biotechnology, University of Wrocław, Wrocław, Poland; ^2^ Laboratory of Protein Mass Spectrometry, Faculty of Biotechnology, University of Wrocław, Wrocław, Poland

**Keywords:** *Porphyromonas gingivalis*, CRP/FNR superfamily, CRP, gene expression regulation, biofilm, virulence

## Abstract

**Introduction:**

*Porphyromonas gingivalis* encodes three CRP/FNR superfamily proteins: HcpR, PgRsp, and Crp^Pg^, with Crp^Pg^ similar to cAMP-sensing proteins but not classified into known families. This study investigates the role of Crp^Pg^ in regulating the expression of factors essential for *P. gingivalis* virulence in A7436 and ATCC 33277 strains.

**Methods:**

The role of Crp^Pg^ protein in *P. gingivalis* was determined using the Δ*crp^Pg^* mutant strains to characterize their phenotype and to assess the impact of *crp^Pg^* inactivation on gene expression using RNA-seq and RT-qPCR. Additionally, the Crp^Pg^ protein was purified and characterized.

**Results:**

Key findings in the Δ*crp^Pg^* mutant strain include up-regulated *mfa1-5* and *rgpA* genes and down-regulated *trxA, soxR*, and *ustA* genes. While *crp^Pg^* inactivation does not affect growth in liquid culture media, it impairs biofilm formation and enhances adhesion to and invasion of gingival keratinocytes. Crp^Pg^ binds directly to its own and *mfa* promoters without interacting with cyclic nucleotides or di-nucleotides. Its three-dimensional structure, resembling *E. coli* Crp in complex with cAMP and DNA, suggests that Crp^Pg^ functions as a global regulator independently of cAMP binding. The highest *crp^Pg^* expression in the early exponential growth phase declines as cell density and metabolic conditions change over time, suggesting a regulatory function depending on the Crp^Pg^ protein amount.

**Conclusions:**

By controlling the shift from planktonic to biofilm lifestyle, Crp^Pg^ may play a role in pathogenicity. Regulating the expression of virulence factors required for host cell invasion and intracellular replication, Crp^Pg^ may help *P. gingivalis* evade immune responses.

## 1 Introduction

CRP/FNR (cyclic AMP receptor protein/fumarate and nitrate reductase regulatory protein) superfamily proteins are widespread among bacteria and act as global regulators controlling large regulons or as more specialized transcription factors (Matsui et al., 2013; Krol et al., 2023; Korner et al., 2003). The type of genes regulated by these proteins depends on the species and often the strain and the environment in which the bacteria live. Members of this superfamily respond to a broad spectrum of intracellular and exogenous signals, such as cyclic nucleotides and di-nucleotides, nitric oxide, carbon monoxide, redox conditions, oxidative and nitrosative stress, temperature, nutrient availability, and quorum sensing. They usually respond to signals by binding allosteric effector molecules or through prosthetic groups interacting with signals, which leads to the activation or repression of gene expression. Among them are genes important for energy metabolism, biofilm formation, antibiotic resistance, and production of virulence factors (Stelling et al., 2010; Smith et al., 2017; Akhter et al., 2007; Śmiga and Olczak, 2019; Fazli et al., 2011; Zheng et al., 2004).

The best-characterized member of the CRP/FNR superfamily is a Crp protein (also known as catabolite gene activator protein, Cap), a key bacterial regulator controlling a variety of biological functions, including carbon metabolism pathway and activity of enzymes engaged in amino acid metabolism (Gosset et al., 2004; Pal et al., 2022; Deutscher, 2008; Kochanowski et al., 2021). *Escherichia coli* Crp (Crp^Ec^) serves as a model bacterial transcription factor. Three-dimensional protein structures of both apo-Crp^Ec^ and Crp^Ec^-cAMP complex are known (e.g., Passner and Steitz, 1997; Sharma et al., 2009; Seok et al., 2014; Youn and Carranza, 2023). This protein is functional in a homodimeric form composed of two subunits, each possessing a typical helix-turn-helix (HTH) motif. Both domains are connected by the C-helix forming a dimerization interface. Classical Crp senses change in cAMP concentration *via* the formation of a cAMP-Crp complex, which results in stable dimer formation and the movement of the regulatory domain that allows its subsequent binding to target DNA sequences, finally leading to inhibition or activation of DNA-binding ability (Sharma et al., 2009; Won et al., 2000). Bacteria often encode more than one homolog of CRP/FNR superfamily proteins; however, those proteins are classified into different families, therefore playing different functions (Mesa et al., 2006; Agari et al., 2008, 2012; Zhou et al., 2012; Yang et al., 2016; Meyer et al., 2018).

*P. gingivalis* is a Gram-negative, anaerobic, black-pigmented, opportunistic pathogen involved in dysbiosis in the oral microbiome and the development of periodontitis (Bostanci and Belibasakis, 2012; Deng et al., 2017; Hajishengallis and Diaz, 2020). The bacterium is often associated with the onset and progression of inflammation-based comorbidities (Arimatsu et al., 2014; Dominy et al., 2019; Mei et al., 2020; Read et al., 2021; Bregaint et al., 2022). *P. gingivalis* can survive not only in the oral cavity as a constituent of a biofilm and dental plaque and inside host cells, but it is also able to spread and survive in other host niches (Kobayashi et al., 2020; Schmidt et al., 2019). Since *P. gingivalis* is an asaccharolytic, non-fermenting bacterium and a heme auxotroph, it developed efficient mechanisms to survive in a hostile environment of the human host. The bacterium produces proteolytic enzymes, including extremely active lysine-specific (Kgp) and arginine-specific (RgpA and RgpB) gingipains involved in the degradation of host proteins enabling the use of peptides as an energy source (Guo et al., 2010; Nemoto and Ohara-Nemoto, 2016). Since *P. gingivalis* does not produce and utilize iron chelators (siderophores or xenosiderophores) and does not possess a functional pathway of heme (iron protoporphyrin IX, FePPIX) biosynthesis, the bacterium must acquire heme as an iron and PPIX source (Smalley and Olczak, 2017; Olczak et al., 2024).

The need to survive in the changing conditions of the host, and importantly to provoke the disease, forces the need for rapid sensing of changing conditions and subsequent efficient adaptation. Therefore, metabolic pathways in bacteria must be under strict and regulated control. The most important bacterial players involved in cell metabolic control are transcription factors. *P. gingivalis* uses several gene expression regulators, forming a multi-layer regulatory network. Some of *P. gingivalis* transcription factors have been characterized, the best example being a homolog of the ferric uptake regulator, PgFur (Śmiga et al., 2019a, b; Ciuraszkiewicz et al., 2014; Butler et al., 2014, 2015), LuxR (Chawla et al., 2010), HaeR (Scott et al., 2013), PorX (Schmitz et al., 2022), and other regulators (Ota et al., 2017; Lewis et al., 2012; Dou et al., 2016, 2018; Boutrin et al., 2016; Śmiga and Olczak, 2019; Qiu et al., 2023). However, in contrast to other bacteria, the function of many of them in *P. gingivalis* is still not known.

*P. gingivalis* encodes 3 homologs of CRP/FNR superfamily proteins. Two of them (HcpR and PgRsp) have been characterized: HcpR responds to nitrosative stress (Lewis et al., 2012), whereas PgRsp is a redox sensing protein that regulates the bacterial response to changing redox conditions (Śmiga and Olczak, 2019). Interestingly, both proteins bind heme (Belvin et al., 2019a, b; Śmiga and Olczak, 2019), most likely used for signal sensing. The role of the third homolog annotated as a Crp protein (locus ID PG0396 in the W83 reference strain) is unknown, and our previous phylogenetic analysis did not classify this protein into any known families (Śmiga and Olczak, 2019). Based on its three-dimensional protein structure (PDB ID: 2GAU) similar to the Crp homologous proteins, we ascribed it as Crp^Pg^. This study aimed to analyze the properties of the Crp^Pg^ protein and identify the processes it may regulate in the context of *P. gingivalis* virulence.

## 2 Materials and methods

### 2.1 Bacterial strains and growth conditions

*P. gingivalis* A7436 and ATCC 33277 (33277) wild-type strains, *crp^Pg^* gene deletion mutant strains (A7436Δ*crp^Pg^* and 33277Δ*crp^Pg^*), and complemented strain constructed in the A7436 genetic background (Δ*crp^Pg^* +Crp^Pg^-HA) (**Supplementary Table S1**, **Supplementary Figure S1**) were grown anaerobically at 37°C (Whitley A35 anaerobic workstation; Bingley, UK) on Schaedler blood agar (ABA) plates (Biomaxima, Lublin, Poland) as previously described (Śmiga et al., 2023). After 3-5 days of culturing on ABA plates, bacteria were transferred into a basal medium (BM) composed of 3% tripticase soy broth (Becton, Dickinson, Sparks, MD, USA) and 0.5% yeast extract (Biomaxima, Lublin, Poland), supplemented with 0.5 µg/ml menadione (Fluka, Munich, Germany), 0.05% cysteine (Sigma-Aldrich, St. Louis, MO, USA), and 7.7 µM hemin chloride (Fluka), resulting in iron and heme-replete conditions (Hm medium). To starve bacteria from iron and heme, BM medium was supplemented with 160 μM 2,2-dipyridyl (Sigma-Aldrich) to chelate iron, and hemin chloride was not added, resulting in iron and heme-depleted conditions (DIP medium). Respective strains were grown with appropriate antibiotics (**Supplementary Table S1**). In most experiments, planktonic bacteria were maintained under anaerobic conditions in culture tubes. To assess the growth curves, bacterial growth was monitored by measuring optical density at 600 nm (OD_600_) over time every 1 h using 96-well plates and a Stratus plate reader (Cerillo, Charlottesville, VA, USA).

*Streptococcus gordonii* ATCC 10558 and *Prevotella intermedia* 17 were cultured as described previously (Śmiga et al., 2015; Ślęzak et al., 2020). *E. coli* Rosetta 2 (DE3) was cultured under standard aerobic conditions.

### 2.2 Generation of modified *P. gingivalis* strains

A7436Δ*crp^Pg^* and 33277Δ*crp^Pg^* mutant strains were generated by replacing the majority of the *crp^Pg^* gene in A7436 and 33277 wild-type strains with erythromycin resistance cassette from *Bacteroides fragilis* (*ermF*) (**Supplementary Figure S1**, **Supplementary Table S1**). Briefly, the flanking regions of the *crp^Pg^* gene and the *ermF* gene were amplified by PCR and primers listed in **Supplementary Table S2**. The obtained fragments were ligated using the NEBuilder HiFi DNA Assembly Cloning Kit (New England Biolabs, Ipswich, MA, USA), resulting in the linear DNA construct that was introduced into *P. gingivalis* A7436 and 33277 strains by electroporation (Simpson et al., 2000). Selection of mutants was carried out on ABA plates supplemented with 3 µg/ml erythromycin (Carl Roth, Karlsruhe, Germany) and homologous recombination between linear construct and chromosomal DNA was verified using PCR, RT-PCR (**Supplementary Figure S1**), and DNA sequencing (Microsynth Seqlab GmbH, Gottingen, Germany).

To generate A7436Δ*crp^Pg^* complemented strain (Δ*crp^Pg^* +Crp^Pg^-HA), producing Crp^Pg^ protein with HA-tag at the C terminus, the *crp^Pg^* gene with its native promoter was amplified by PCR and primers listed in **Supplementary Table S2**. Then, the DNA fragment was cloned into XhoI and BamHI restriction sites of a pTIO-tetQ plasmid, resulting in a pTIO-tetQ+crp^Pg^ plasmid. pTIO-tetQ plasmid was obtained from the original pTIO-1 plasmid (Tagawa et al., 2014) by replacing the *ermF* resistance cassette with the *tetQ* resistance cassette (Śmiga et al., 2019a). The cloned DNA sequence was verified by DNA sequencing (Microsynth Seqlab). The pTIO-tetQ+crp^Pg^ plasmid was introduced into *P. gingivalis* A7436Δ*crp^Pg^* mutant strain by electroporation. Selection of a Δ*crp^Pg^* +Crp^Pg^-HA strain was carried out on ABA plates supplemented with 2 µg/ml tetracycline (Carl Roth). The *crp^Pg^* gene expression was verified at transcript and protein levels using RT-PCR and Western blotting, respectively (**Supplementary Figure S1**).

### 2.3 Overexpression and purification of recombinant proteins

To overexpress and purify Crp^Pg^ protein with an N-terminal tag (6×His and maltose-binding protein; 6×His-MBP), the *P. gingivalis* A7436 *crp^Pg^* gene (PGA7_RS07240) was amplified using PCR and primers listed in **Supplementary Table S2** and cloned into XmnI and BamHI restriction sites of pMAL c5x_His plasmid (Śmiga et al., 2019a). DNA sequencing (Microsynth) was carried out to verify the cloned DNA sequence.

Recombinant Crp^Pg^ protein in fusion with N-terminal 6×His-MBP tag was overexpressed in *E. coli* Rosetta 2 (DE3) strain (Sigma-Aldrich). After heat-shock transformation, bacteria were grown in terrific broth (TB) with the addition of 35 µg/ml chloramphenicol (Carl Roth) and 100 µg/ml carbenicillin (A&A Biotechnology, Gdansk, Poland) (37°C, 220 rpm) until OD_600_~0.8 was reached. Protein overexpression was induced by IPTG (Carl Roth) added to the final concentration of 0.5 mM, and the culture was incubated for 16 h at 16°C with shaking (220 rpm). Then, bacteria were centrifuged (4000×*g*, 20 min, 4°C) and the pellet was kept at -20°C until needed. Bacteria were suspended in 25 mM HEPES, pH 7.8, containing 300 mM NaCl and lysed with sonication (Sonopuls HD 4100; Bandelin, Berlin, Germany). Next, bacterial lysates were centrifuged (30000×*g*, 20 min, 4°C). 6×His-MBP-Crp^Pg^ recombinant protein was purified from the soluble fraction using TALON Superflow resin (Sigma). After removing the unbound fraction, the resin was washed with 25 mM HEPES, pH 7.8, containing 300 mM NaCl and 5 mM imidazole, followed by 25 mM HEPES, pH 7.8, containing 1 M NaCl and 5 mM imidazole. 6×His-MBP-Crp^Pg^ protein was eluted with 25 mM Tris-HCl, pH 7.0, containing 250 mM NaCl and 150 mM imidazole. Finally, the purified recombinant 6×His-MBP-Crp^Pg^ protein was subjected to proteolytic cleavage using Factor Xa in the presence of 2 mM CaCl_2_ (New England Biolabs) for 48-72 h at 4°C. Released 6×His-MBP fusion protein was bound to amylose resin according to the manufacturer’s protocol (New England Biolabs). Purified Crp^Pg^ protein was concentrated using Amicon^®^ Ultra Centrifugal Filter (10 kDa MWCO; Millipore, Billerica, MA, USA) and stored in 25 mM Tris-HCl buffer, pH 7.0, containing 250 mM NaCl and 10% glycerol at -20°C until used. To measure Crp^Pg^ protein concentration, the empirical molar absorption coefficient was determined (ϵ_280_ = 22514 M^-1^ cm^-1^) as reported by others (Eakanunkul et al., 2005).

The PgRsp protein was overexpressed and purified as previously reported (Śmiga and Olczak, 2019).

### 2.4 *P. gingivalis* interaction with host cells

The ability of *P. gingivalis* to interact with immortalized human oral gingival keratinocytes (Gie-No3B11; ABM, Richmond, British Columbia, Canada) was analyzed as described previously (Śmiga et al., 2023). Keratinocytes were maintained in TM040 medium (ABM), supplemented with 2% heat-inactivated fetal bovine serum (FBS; Cytogen, Zgierz, Poland), 100 U/mL penicillin and 100 µg/mL streptomycin (Cytogen) at 37°C in an atmosphere of 5% CO_2_. Cells were washed three times with PBS and suspended in a DMEM medium (Sigma Aldrich, Cat. No. D0822) without added serum and antibiotics. Bacteria cultured for 24 h in Hm medium were centrifuged (4000×*g*, 20 min, 4°C) and washed with PBS. Keratinocytes were incubated for 4 h at 37°C in an atmosphere of 5% CO_2_ with *P. gingivalis* at the multiplicity of infection (MOI) of 100. The medium was collected, wells were washed three times with PBS, and a fresh DMEM medium was added to determine the bacteria present inside the cells and attached to them. To kill the external bacteria and to determine live bacteria inside the cells only, the DMEM medium was supplemented with 300 μg/mL gentamicin (Sigma-Aldrich) and 200 μg/mL metronidazole (Sigma-Aldrich). After 1 h, the wells were washed three times with PBS, and the cells were lysed with sterile distilled water. The cell lysates were used to prepare serial dilutions and subsequently plated on ABA plates. The plates were incubated for 7–10 days at 37°C under anaerobic conditions to determine colony-forming units (CFU/1 mL). The experiment was carried out at least three times using two independent biological samples, each sample examined in two technical repetitions.

### 2.5 Determination of gingipain activity

Rgp and Kgp activities were measured as reported by others (Pomowski et al., 2017). Briefly, to 100 µl of 50 mM Tris-HCl buffer, pH 7.5, containing 150 mM NaCl (TBS), supplemented with 0.05% Tween 20, 5 mM CaCl_2_ (TTBS), containing 10 mM L-cysteine hydrochloride, the latter neutralized with 10 mM NaOH, 10 µl of *P. gingivalis* whole cultures was added. Samples were incubated for 10 min at 37°C and the reaction was initiated by adding 100 µl of TTBS, supplemented with 1 mM Nα-Benzoyl-DL-arginine p-nitroanilide hydrochloride (BApNA; Sigma-Aldrich) for Rgp or N-(p-tosyl)-Gly-Pro-Lys 4-nitroanilide acetate salt (Sigma-Aldrich) for Kgp activity measurement. Samples were incubated for 2 h at 37°C and the reaction was monitored by measuring the absorbance at 405 nm over time using a GloMax Discover plate reader (Promega). Gingipain activity was normalized to the OD_600_ of the bacterial culture and compared to the wild-type strains’ activity, the latter set as 100%.

### 2.6 Biofilm formation

Biofilm formation was analyzed using 96-well plates (Corning, NY, USA) or plates coated with *S. gordonii* or *P. intermedia*. A fresh Hm medium was inoculated with *S. gordonii* or *P. intermedia* to the OD_600_ = 0.3. Then, 100 μl of bacterial cultures were added per well and incubated at 37°C under anaerobic conditions for 24 h to create a biofilm composed of *S. gordonii* or *P. intermedia*. Unbound bacteria were washed 3 times with 200 µl of TBS. *P. gingivalis* was grown for 24 h in Hm medium, centrifuged (4000×*g*, 20 min, 20°C), and washed with TBS. Then, bacteria were resuspended in TBS to OD_600_ = 2, and 100 μl of bacterial suspension was added per well pre-coated with *S. gordonii* or *P. intermedia*, or directly to uncoated wells. Samples were incubated for 1 h at 37°C and unbound bacteria were washed 3 times with TBS. Biofilm formation was visualized using 3 methods:

#### 2.6.1 Crystal violet staining

50 μl of 1% crystal violet solution (Carl Roth) was added to wells and incubated for 15 min. The solution was poured off and the wells were washed 5 times with TBS. Then, 100 μl of 99.9% ethanol was added to wells, incubated for 5 min, and mixed by pipetting. Next, the absorption at 560 nm (A_560_) was measured by a GloMax Discover plate reader (Promega).

#### 2.6.2 Determination of Kgp activity

The Kgp activity was determined as described above for whole bacterial cultures. In this experiment, we determined the proteolytic activity of the membrane-bound Kgp only. Since the substrate can penetrate biofilm structure, we assumed that Kgp activity (ΔA_405nm_/60 min) is proportional to the number of *P. gingivalis* cells forming a biofilm.

#### 2.6.3 Determination of HmuY protein amount

HmuY protein was determined using anti-HmuY antibodies (Śmiga et al., 2015). The biofilm was fixed using 50 μl of 4% paraformaldehyde (Thermo Fisher Scientific) in 20 mM sodium phosphate buffer, pH 7.4, containing 140 mM NaCl (phosphate buffered saline, PBS) for 10 min, washed 3 times with 200 μl of TBS and blocked with 200 µl of 2% bovine serum albumin (BSA, Carl-Roth) in TBS. After 1-h incubation at 37°C, 100 μl of 10000× diluted anti-HmuY antibodies in TBS, supplemented with 0.1% BSA was added and incubated overnight at 4°C. Then, wells were washed 4 times with 200 μl of TBS and 100 μl of goat anti-rabbit IgG antibodies conjugated with HRP (Sigma-Aldrich) in TBS, supplemented with 0.1% BSA (1:10000) was added and the samples were incubated for 1 h at 37°C. After 4 final washes with TBS, HmuY protein was visualized using 100 µl of 0.05% *o*-phenylenediamine (Sigma-Aldrich) in buffer composed of 48.5 mM citric acid and 103 mM Na_2_HPO_4_, pH 5.0, supplemented with 0.01% H_2_O_2_. The samples were incubated at room temperature for 10 min and the reaction was stopped by adding 25 μl of 12.5% H_2_SO_4_. Absorbance at 450 nm (A_450_) was measured using a GloMax Discover plate reader (Promega). In this experiment, we determined the amount of membrane-bound HmuY protein, potentially in *P. gingivalis* located on the surface of the biofilm.

### 2.7 Determination of phosphodiesterase activity

PDE activity was determined as previously reported (Lin et al., 2016) with minor modifications. Briefly, *P. gingivalis* was grown to the early stationary phase (~24 h) in Hm medium, centrifuged (4000×*g*, 20 min, 20°C), and washed with TBS. Bacteria were suspended in TBS, supplemented with a protease inhibitor cocktail (Bimake, Houston, TX, USA), and stored at -80°C. Bacterial samples were thawed and lysed by sonication (Bandelin). The protein content was measured in the lysates using Roti Nanoquant (Carl Roth, Karlsruhe, Germany). For the PDE activity measurement, the samples containing 3 µg of proteins in 50 mM Tris-HCl buffer, pH 8.5, supplemented with 1 mM MnCl_2_ were placed in a 96-well plate. The reaction was started by adding bis(*p*-nitrophenyl) phosphate sodium salt (Sigma-Aldrich) at the final concentration of 5 mM. The samples were incubated at 37°C and the absorbance at 405 nm (A_405_) was measured over time using a GloMax Discover plate reader (Promega). PDE activity was determined as the change in absorbance at 405 nm over 60 min (ΔA_405nm_/60 min).

### 2.8 Determination of ATP/ADP and NAD^+^/NADH contents

Nucleotide contents were determined in *P. gingivalis* grown for 6 and 24 h in Hm medium. Samples containing approximately 2×10^9^ bacterial cells (1 ml of bacterial culture at OD_600_ equal to 2) were centrifuged (6000×*g*, 10 min, 4°C), washed once with PBS, and stored at -80°C.

To determine ATP and ADP contents, the luminescence-based ADP/ATP Ratio Assay Kit (Sigma-Aldrich, catalog no: MAK135) was used. The bacterial pellets were suspended in 200 µl of PBS supplemented with 0.2% SDS, incubated for 10 min at 60°C, and then subjected to the procedure provided by the manufacturer.

To determine NAD+ and NADH contents, the colorimetric-based NAD/NADH Assay Kit (Sigma-Aldrich, catalog no: MAK468) was used according to the manufacturer’s protocol. Lysis of bacterial pellets was performed using buffers supplied by the manufacturer.

### 2.9 Gene expression analysis using reverse transcriptase-quantitative polymerase chain reaction

RNA isolation, reverse transcriptase reaction (RT), and quantitative PCR (qPCR) were performed as previously described (Śmiga et al., 2023). Relative quantification of the transcript was calculated using the double delta method (ΔΔCq) and *P. gingivalis 16S rRNA* as a reference gene. All primers used in this study are listed in **Supplementary Table S2**. At least three independent experiments were run in triplicate for the target and reference genes.

### 2.10 Determination of conditions affecting *crp^Pg^* expression

#### 2.10.1 influence of the growth phase

To analyze the effect of the growth phase on the expression of the *crp^Pg^* gene, total RNA was isolated from bacteria grown in Hm medium for 4 h (early exponential growth phase), 10 h (late exponential growth phase), and 24 h (stationary growth phase).

#### 2.10.2 influence of keratinocyte extracellular components

Cell-free culture medium collected after 24 h (DMEM^KER^) from keratinocyte cultures was used to examine the influence of extracellular components produced by host cells on *P. gingivalis crp^Pg^* expression. Briefly, DMEM^KER^ was collected, centrifuged for 5 min (400×g, room temperature), and filtered using 0.22 µM sterile syringe filters (Carl Roth). Overnight *P. gingivalis* cultures in Hm medium were used to inoculate the fresh DMEM or DMEM^KER^ culture media at a starting OD_600_ = 0.1 and maintained under anaerobic conditions at 37°C for 4 h. RNA was isolated from bacteria collected from 2-ml cultures.

### 2.11 Gene expression analysis using RNA-sequencing

*P. gingivalis* was grown to the mid-exponential growth phase (OD_600_ = 0.5-0.6). Then, bacteria were pelleted (6000×*g*, 10 min, 4°C), suspended in 20 µl of 75% ethanol, immediately frozen in liquid nitrogen, and stored at -80°C. RNA isolation, sequencing, and gene expression analysis were performed by Novogene (Cambridge, United Kingdom) using standard company protocols. Gene expression experiment was performed using three independent biological replicates. Fold changes >1.5 or <-1.5 with adjusted *P* value (P_adj_) <0.05 were considered significant. The expression of selected genes was validated using RT-qPCR with total RNA extracted from the same bacterial cultures used for RNA-seq analysis.

### 2.12 Sodium dodecyl sulfate-polyacrylamide gel electrophoresis and western blotting

Bacterial lysates were prepared with a protease inhibitor cocktail (Bimake) and standardized to OD_600_ or to the amount of protein determined using Roti Nanoquant (Carl Roth). Samples were separated using SDS-PAGE and either stained with Coomassie Brilliant Blue G-250 (CBB G-250) or transferred onto nitrocellulose membranes (Millipore), and probed with mouse anti-HA (0.5 µg/ml; Thermo Fisher Scientific, Waltham, MA, USA), rabbit anti-HmuY (1:10000) (Śmiga et al., 2015), or rabbit anti-RgpB (0.5 µg/ml) (Cusabio, Houston, TX, USA) antibodies. The complexes were further probed with goat horseradish peroxidase (HRP)-conjugated anti-mouse (1:10000; Promega, Madison, WI, USA) or anti-rabbit (1:10000; Sigma-Aldrich) IgG antibodies. Alternatively, the membranes were incubated with lectins (Vector Laboratories, Newark, CA, USA) as previously described (Śmiga et al., 2019b, 2024a). Formed complexes were visualized using chemiluminescence staining (PerkinElmer, Waltham, MA, USA or Thermo Fisher Scientific) and the ChemiDoc imaging system (Bio-Rad, Hercules, CA, USA).

### 2.13 Protein identification by mass spectrometry

The ~100 kDa and ~120 kDa gel sections were excised from electrophoretic lanes corresponding to samples from 33277 and 33277Δ*crp^Pg^*. Sections were then prepared for bottom-up ESI-LC-MS. Analyses were carried out on an M-class Acquity nanoUPLC connected to a Synapt XS HDMS (Waters, Milford, MA, USA), and the data was analyzed with the PLGS v3.0.3 software. Sample preparation, LC-MS conditions, and data processing protocols were described in detail in our previous work (Śmiga et al., 2024b). For the glycopeptide search via oxonium ion identification, the N- and O-linked Variable Glycosylation Modification was added to the PLGS search workflow.

### 2.14 Analysis of Crp^Pg^ dimerization

The formation of protein dimers was analyzed using cross-linking of Crp^Pg^ with formaldehyde (Sigma-Aldrich). 10 μM Crp^Pg^ was incubated in PBS for 30 min at room temperature. Alternatively, 1 mM cAMP was added to the sample. Then, samples were incubated for 1 h at 37°C with 0.2% formaldehyde. To visualize dimers, all samples were analyzed using SDS-PAGE and CBB G-250 staining.

### 2.15 Analysis of nucleotide binding

To examine whether the Crp^Pg^ protein binds cyclic nucleotides or di-nucleotides, the affinity chromatography with selected nucleotides immobilized on agarose resin was used: 2-AHA-cAMP agarose, 8-AHA-cAMP-agarose, 5’AHC-2’,3’-cAMP-agarose, 2’-AHC-cAMP-agarose, 2’-AHC-cGMP-agarose, 2-AH-cGMP-agarose, 2’-AHC-cCMP-agarose, 4-AH-cCMP-agarose, 2’-AHC-c-di-GMP-agarose, 2’-AHC-c-di-AMP-agarose (Biolog Life Science Institute, **Supplementary Figure S2**). 5 μM Crp^Pg^ protein in 25 mM Tris-HCl buffer, pH 7.0, containing 250 mM NaCl and 5 mM MgCl_2_. The protein solution was added to the appropriate resin, incubated for 30 min, and samples were centrifuged (500×*g*, 2 min). To wash out unbound protein, the resin was incubated for 10 min with an initial buffer and centrifuged. The protein-binding process was monitored by SDS-PAGE and staining with CBB G-250.

In addition, the binding of the purified Crp^Pg^ to fluorescent cAMP analog (2-Aza-ε-cAMP; Biolog Life Science Institute) was analyzed. Briefly, 50 µM 2-Aza-ε-cAMP was added to the 20 µM protein solution and the sample was incubated for 1 h at room temperature. Alternatively, 250 μM cAMP was additionally added to the sample for competitive binding with 2-Aza-ε-cAMP. To verify whether the protein can bind the 2-Aza-ε-cAMP, unbound nucleotide was removed using Amicon Ultra-4 Centrifugal Ultracel-10KDa filter units (Millipore) and washing once with PBS. After the final concentration, the sample volume was adjusted to the initial volume, and 200 µl of the obtained sample was used for fluorescence measurement using a GloMax Discover plate reader (Promega) with a wavelength of excitation and emission set at 365 nm and 500–550 nm, respectively.

### 2.16 Electromobility shift assay

The LightShift Chemiluminescent EMSA Kit (Thermo Fisher Scientific) was used to examine Crp^Pg^ binding to the selected DNA probes according to the manufacturer’s protocol. Biotin-labeled DNA fragments were amplified by PCR using primers listed in **Supplementary Table S2**. Purified biotin-labeled DNA probes (1 ng for the entire promoters sequences or 0.33 ng for promoters’ fragments per sample) were added to the binding buffer, containing 2.5% glycerol, 5 mM MgCl_2_, 50 ng/μL poly (dI-dC), 50 mM KCl, and 0.05% NP-40. Alternatively, various concentrations of the purified Crp^Pg^ protein, non-biotinylated DNA (100 ng for the entire promoter or 33 ng for shorter fragments per sample), and 1 mM nucleotides (cAMP, cGMP, c-di-AMP, c-di-GMP) were added. Samples were incubated for 20 min at room temperature and then subjected to electrophoresis on pre-run (for 30 mins) 6% polyacrylamide gels for 80 min at 200 V in 0.25× TBE buffer (1× TBE buffer, pH 8.6, contains 25 mM Tris, 25 mM boric acid, 0.5 mM EDTA). Subsequently, samples were transferred onto a nylon membrane (Bionovo, Legnica, Poland) by wet transfer in 0.5× TBE buffer for 30 min at 380 mA and cross-linked for 10 min using UV radiation at 254 nm. Labeled DNA was visualized using the Chemiluminescent Nucleic Acid Detection Module Kit (Thermo Fisher Scientific) and the ChemiDoc MP imaging system (Bio-Rad).

### 2.17 Bioinformatics analyses

Phylogenetic analyses and search for Crp^Pg^ (accession number WP_005873831) homologous proteins were performed using BLASTP (https://www.ncbi.nlm.nih.gov/). Protein sequence comparison was performed using The Clustal Omega (Madeira et al., 2022), Sequence Manipulation Suite: Ident and Sim (Stothard, 2000), and Jalview (Waterhouse et al., 2009). The phylogenetic tree was created using Simple Phylogeny (Madeira et al., 2022) and visualized using iTOL (Letunic and Bork, 2021). Theoretical Crp^Pg^ function was predicted using I-TASSER (https://zhanggroup.org/), COACH (Yang et al., 2013a, b), COFACTOR (Zhang et al., 2017; Roy et al., 2012), and EDock (Zhang et al., 2020) tools based on the known Crp^Pg^ three-dimensional structure (PDB ID: 2GAU) and amino acid sequence (accession number WP_005873831). Visualization of protein structures was performed with UCSF Chimera (https://www.cgl.ucsf.edu/chimera) (Pettersen et al., 2004).

### 2.18 Statistical analysis

All experiments were conducted at least three times using a minimum of three biological replicates, with each sample tested in technical replicates. Unpaired Student’s *t*-test, one-way analysis of variance (ANOVA) with *post hoc* Tukey’s test, or two-way ANOVA with *post hoc* Sidac test (to analyze biofilm formation) was applied using GraphPad software (GraphPad Prism 8.0 Inc., San Diego, CA, USA). All results are shown as mean ± standard deviation (mean ± SD), and for analysis of growth curves as mean ± standard error (mean ± SE).

## 3 Results

### 3.1 Assignment of Crp^Pg^ protein to the CRP/FNR superfamily

**Figure 1:**
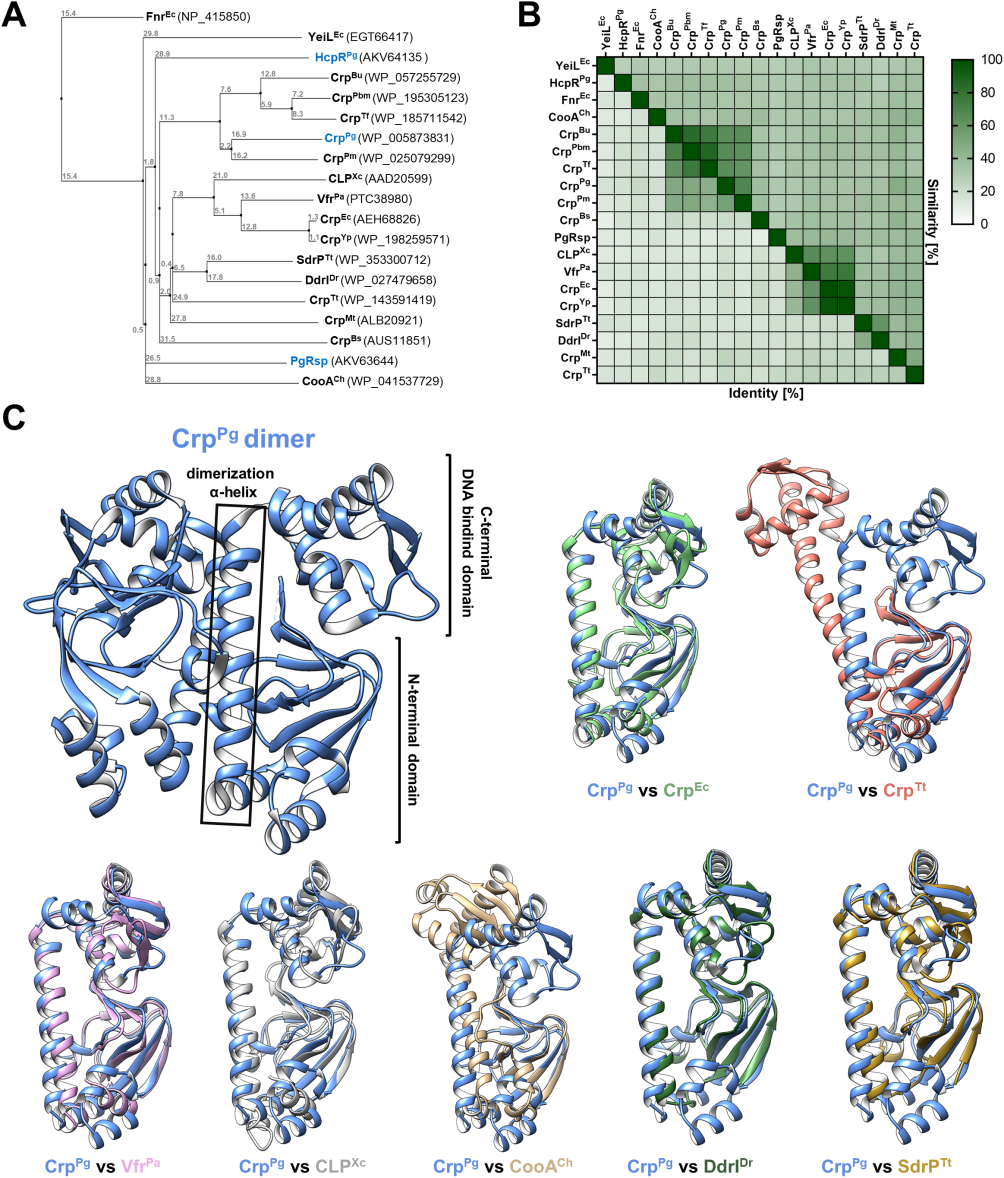
Theoretical structure-function analysis of *P. gingivalis* Crp^Pg^. **(A)** A phylogenetic tree was generated using selected amino acid sequences of proteins from the CRP/FNR superfamily based on % identity (PID). Crp/Fnr protein homologs from *Porphyromonas gingivalis* are shown in blue. **(B)** Heat map showing % identity and similarity between protein sequences of selected CRP/FNR superfamily representatives. **(C)** The three-dimensional structure of the Crp^Pg^ protein (PDB: 2GAU) shows the typical Crp protein regions, including the N-terminal domain involved in ligand binding, the C-terminal DNA-binding domain, and the α-helix connecting both domains. Comparison of Crp^Pg^ monomer structure with structures of selected proteins from the CRP/FNR superfamily: Crp from *Escherichia coli* (Crp^Ec^, PDB: 2GZW), Crp from *Thermus thermophilus* (Crp^Tt^ PDB: 4EV0), Vfr from *Pseudomonas aeruginosa* (Vfr^Pa^, PDB: 2OZ6), CLP from *Xanthomonas campestris* (CLP^Xc^, PDB: 3IWZ), CooA from *Carboxydothermus hydrogenoformans* (CooA^Ch^, PDB: 2FMY), DdrI from *Deinococcus radiodurans* (DdrI^Dr^, PDB: 8YZ7), and SdrP from *T. thermophilus (*SdrP^Tt^, PDB: 2ZCW). Bu, *Bacteroides uniformis*; Pbm, *Parabacteroides merdae*; Tf, *Tannerella forsythia*; Pm, *Porphyromonas macacae*; Yp, *Yersinia pestis*; Mt, *Mycobacterium tuberculosis*; Bs, *Bacillus subtilis*; PgRsp, *P. gingivalis* redox-sensing protein; HcpR, Fnr-like protein from *P. gingivalis*.

Preliminary theoretical analyses (Śmiga and Olczak, 2019) suggested that the protein (accession number WP_005873831) encoded in *P. gingivalis* A7436 and 33277 strains by *PGA7_RS07240* and *PGN_1569* genes, respectively (*PG0396* gene in the reference *P. gingivalis* W83 strain), showed homology to proteins from the CRP/FNR superfamily. However, our previous phylogenetic analysis indicated that this protein is not grouped within families comprising proteins with known functions (Śmiga and Olczak, 2019). An updated phylogenetic analysis carried out in this study confirmed and extended the previous findings (**Figure 1A**). BLASTP search demonstrated the close similarity to the uncharacterized proteins encoded within the closely related species from the Bacteroidota phylum, such as *Porphyromonas macacae*, *Parabacteroides merdae*, *Tannerella forsythia*, *Bacteroides uniformis* (**Figures 1A, B**, **Supplementary Figure S3**), with amino acid sequence identity and similarity higher than 43% and 60%, respectively (**Figure 1B**). Comparison of the amino acid sequence of the *P. gingivalis* WP_005873831 protein with selected sequences of characterized Crp proteins showed low identity/similarity: 18.0/34.8% for *Escherichia coli* Crp^Ec^, 18.6/34.8% for *Pseudomonas aeruginosa* Vfr^Pa^, 21.0/41.6% for *Thermus thermophilus* Crp^Tt^, 24.9/46.8% for *Mycobacterium tuberculosis* Crp^Mt^, 20.3/37.0% for *Bacillus subtilis* Crp^Bs^, 17.9/31.7% for *Xanthomonas campestris* CLP^Xc^, 16.6/34.9% for *Deinococcus radiodurans* DdrI^Dr^ (**Figure 1B**). In contrast, the three-dimensional structure of WP_005873831 protein (PDB ID: 2GAU; deposited by Rotella FJ, Zhang RG, Mulligan R, Moy S, and Joachimiak A; Midwest Center for Structural Genomics, MCSG; 2006, revised in 2011), possessing a typical N-terminal ligand-binding domain and a C-terminal DNA-binding domain, is highly similar to other known proteins from the CRP/FNR superfamily (Passner and Steitz, 1997; Agari et al., 2012; Wang et al., 2024). As in homologous proteins, both domains are connected by an α-helix involved in dimerization (**Figure 1C**). Moreover, comparison with proteins of known three-dimensional structures showed the highest structural similarity to Crp proteins (**Figure 1C**). Therefore, we classified *P. gingivalis* protein to the CRP/FNR superfamily and Crp family and termed it as Crp^Pg^.

### 3.2 Crp^Pg^ protein acts as a global regulator

To determine the role of Crp^Pg^ in *P. gingivalis*, we examined two *P. gingivalis* strains, representatives of more (A7436) and less virulent (33277) strains, which differ in phenotypes and virulence potential (Laine and van Winkelhoff, 1998; Griffen et al., 1999; Dorn et al., 2000; Amano et al., 2000; Yoshino et al., 2007; Rodrigues et al., 2012; Ohya et al., 2016; Śmiga et al., 2019b; Seers et al., 2020; Śmiga et al., 2024a). The A7436 strain is encapsulated, produces fewer fimbriae, which is reflected in the formation of smaller biofilm structures and is connected more often with patients with periodontitis. In contrast, the ATCC 33277 strain lacks a capsule, forms larger biofilm structures due to its high fimbriation, and is connected more often with healthy periodontium. In addition, although both strains are fimbrated, they produce different types of the long fimbrial protein FimA (Nagano et al., 2013). Therefore, mutant strains lacking the functional *crp^Pg^* gene were constructed in both A7436 (A7436Δ*crp^Pg^*) and 33277 (33277Δ*crp^Pg^*) strains (**Supplementary Figure S1**).

**Figure 2:**
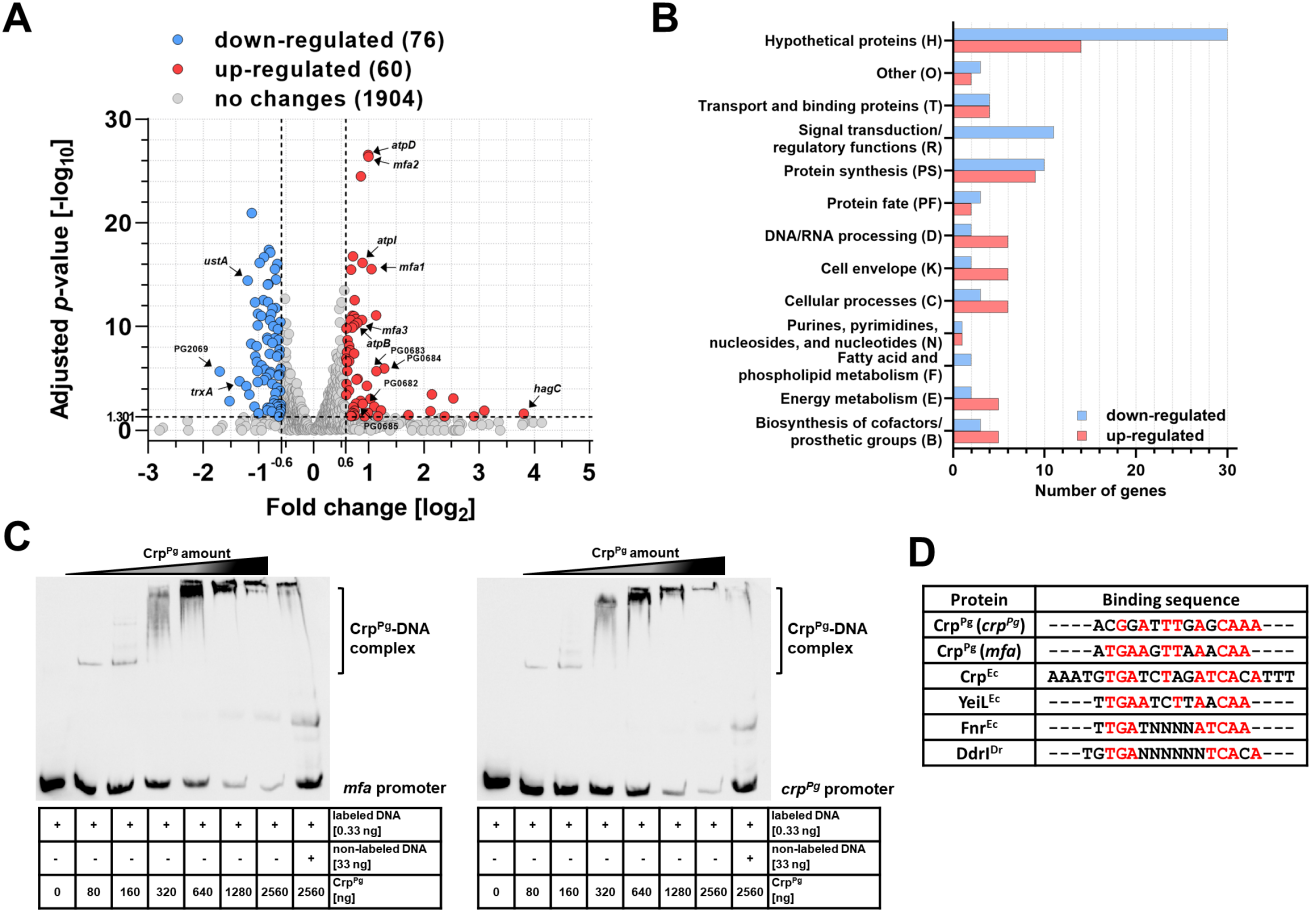
Crp^Pg^ protein acts as a global regulator. **(A)** Summary of RNA-seq data of gene expression in *P. gingivalis* Δ*crp^Pg^* mutant strain shown as a Volcano plot based on RNA-seq results presenting global changes in gene expression of the A7436Δ*crp^Pg^* mutant strain in comparison to the A7436 wild-type strain. Gene expression with fold change < -1.5 (log_2_-0.6) or >1.5 (log_2_0.6) and adjusted *p*-value >0.05 (-log_10_1.301) is considered as significant. Selected gene names are shown in the plot. **(B)** Groups of proteins encoded by genes whose expression has changed significantly. RNA-seq data are shown from three independent biological replicates. **(C)** EMSA was used to determine the binding of the Crp^Pg^ protein to the *mfa* and *crp^Pg^* genes’ promoters. The *crp^Pg^* and *mfa* promoter regions bound by Crp^Pg^ protein were verified using EMSA with increasing protein amount and biotin-labeled promoter regions. In addition, control using 100× fold excess of unlabeled promoter DNA, as well as non-specific DNA competitor (50 ng/μL poly; dI-dC) in all samples were used. The Crp^Pg^-DNA complexes formed are shown as a shift. In addition, protein-DNA aggregates were formed in gel wells suggesting lower non-specific Crp^Pg^ binding to all DNA fragments examined. **(D)** Theoretical DNA fragments recognized by Crp^Pg^ were identified by comparing *crp^Pg^* promoter fragment 4 and *mfa* promoter fragment 5 (**Supplementary Figure S4**) with known sequences recognized by *E. coli* Crp^Ec^, YeiL^Ec^, Fnr^Ec^, and *D. radiodurans* DdrI^Dr^ proteins. The consensus sequence is shown in red.

To examine the influence of Crp^Pg^ protein on the global gene expression, RNA-seq analysis was carried out in the Δ*crp^Pg^* mutant strain constructed in the A7436 genetic background. The expression of selected genes was validated using RT-qPCR. The inactivation of the *crp^Pg^* gene resulted in 76 up-regulated and 60 down-regulated genes (**Figure 2A**). Genes whose expression decreased the most belong mainly to the group of hypothetical proteins, protein synthesis, and signal transduction/regulatory functions (including 5 helix-turn-helix transcriptional regulators) (**Figure 2B**; **Supplementary Table S3**). Among the most highly down-regulated genes in the Δ*crp^Pg^* mutant strain are the genes encoding thioredoxin (TrxA; PG0034) and SDR family oxidoreductase (PG2069) (**Supplementary Table S3**). Also, the expression of the gene encoding a SoxR homolog (SoxR reducing system RseC family protein/positive regulator of sigma(E); PG0302) and two genes encoding 4Fe-4S-binding proteins (PG1421 and PG1813) was decreased (**Supplementary Table S3**). Another highly down-regulated gene encodes up-regulated in stationary phase protein A (UstA; PG0246) (**Table 1**; **Supplementary Table S3**).

**Table 1:** Validation of expression of selected genes in the Δ*crp^Pg^* mutant strain constructed in the A7436 genetic background compared to the A7436 wild-type strain using RT-qPCR.

Gene ID in A7436	Gene ID in ATCC 33277	Gene ID in W83	Gene product description	Gene name	Fold change*	
					RNA-seq	RT-qPCR
PGA7_RS00800	PGN_0287	PG0178	fimbrial major subunit, Mfa1	*mfa1*	2.06	1.46 ± 0.27
PGA7_RS09555	PGN_0180^#^	PG2132	fimbrial major subunit, FimA	*fimA*	-1.19	-1.34 ± 0.43
PGA7_RS05995	not present	PG0683	ABC transporter permease	the gene from the *PG0682*-*PG0685* operon	2.43	2.06 ± 0.58
PGA7_RS01095	PGN_0349	PG0246	up-regulated in stationary phase protein A, UstA	*ustA*	-2.29	-1.33 ± 0.38
PGA7_RS08820	PGN_1906	PG1975	hemagglutinin, HagC	*hagC*	8.55	-1.14 ± 0.06
PGA7_RS08805	PGN_1904	PG1972	hemagglutinin, HagB	*hagB*	-1.01	1.02 ± 0.07
PGA7_RS08180	PGN_1733	PG1837	hemagglutinin, HagA	*hagA*	1.04	-1.41 ± 0.56
PGA7_RS06935	PGN_1503	PG0465	ferric uptake regulator, Fur	*fur*	-1.13	1.02 ± 0.06

^#^Low homology to genes identified in A7436 and W83 strains.

*The experiment was carried out in bacteria grown in iron and heme-replete conditions (Hm medium) and collected in the mid-exponential growth phase (OD_600_ = 0.5-0.6). Positive and negative values indicate increased and decreased gene expression, respectively. Fold change values are shown as a mean for RNA-seq and mean ± standard deviation (mean ± SD) for RT-qPCR determined for 3 biological replicates.

The genes whose expression increased the most belong mainly to the group of hypothetical proteins, protein synthesis, DNA/RNA processing, cell envelope, cellular processes, energy metabolism, biosynthesis of cofactors/prosthetic groups, and transport and binding proteins (**Figure 2B**; **Supplementary Table S3**). Examples of up-regulated genes involved in *P. gingivalis* virulence include those encoding fimbrial Mfa proteins (mainly Mfa1-Mfa3) (PG0178-PG0182) and Arg-specific gingipain A (RgpA; PG2024) (**Table 1**; **Supplementary Table S3**). Among the most highly up-regulated genes in the Δ*crp^Pg^* mutant strain is the *hagC* gene encoding one of the hemagglutinins; however, this result was not confirmed by RT-qPCR analysis (**Table 1**). Using RNA-seq and RT-qPCR analyses we did not observe changes in the expression of other genes encoding hemagglutinins, namely HagA and HagB (**Table 1**). Therefore, these findings strongly suggest that Crp^Pg^ does not regulate the hemagglutinin activity of *P. gingivalis*.

To verify whether the Crp^Pg^ influences the expression of Mfa proteins, the direct Crp^Pg^ interaction with the *mfa* promoter was examined (**Supplementary Figure S4A**; **Supplementary Table S4**). First, a ~250 bp DNA fragment containing the entire promoter was analyzed. After confirmation by EMSA that the Crp^Pg^ binds to this DNA (data not shown), the promoter region was divided into 5 smaller ~85 bp fragments (**Supplementary Figure S4A**). This allowed us to specify the promoter region recognized by the Crp^Pg^ protein (fragment 5 shown in purple in **Supplementary Figure S4B**). As Crp proteins often act as autoregulators (Korner et al., 2003; Śmiga and Olczak, 2019), a similar analysis was performed for the *crp^Pg^* promoter (data not shown) and its fragments (**Supplementary Figure S4**). This allowed us to specify the promoter region recognized by the Crp^Pg^ protein (fragment 4 shown in red in **Supplementary Figure S4B**). Analysis of both selected promoter fragments showed a shift in the migration of DNA (**Figure 2C**; **Supplementary Figure S4B**), indicating DNA-protein complex formation. The Crp^Pg^ also tends to interact with DNA with lower affinity, visible as protein-DNA aggregates formed in the gel wells. Such behavior was also observed in the case of DNA fragments to which the Crp^Pg^ did not bind (e.g., promoters of *sod* and *pgfur*; our unpublished data).

By comparing *mfa* and *crp^Pg^* promoter fragments to those recognized by selected CRP/FNR superfamily proteins it was possible to indicate the potential Crp^Pg^-binding box which showed high similarity to the one bound by *E. coli* Crp^Ec^ (**Figure 2D**). Highly homologous sequences were also found in other promoters, including those for *trxA*, *PG2008*, and *PG0682*-*PG0685* genes (**Supplementary Table S4**) whose expression was changed in the *crp^Pg^* mutant strain (**Supplementary Table S3**). In contrast, similar sequences were identified within the sequences encoding PgFur and HagC, but not in their promoter regions, which may indicate the lack of regulation of the expression of these genes by Crp^Pg^ (**Table 1**; **Supplementary Table S4**).

### 3.3 Crp^Pg^ binding to DNA is ligand-independent

**Figure 3:**
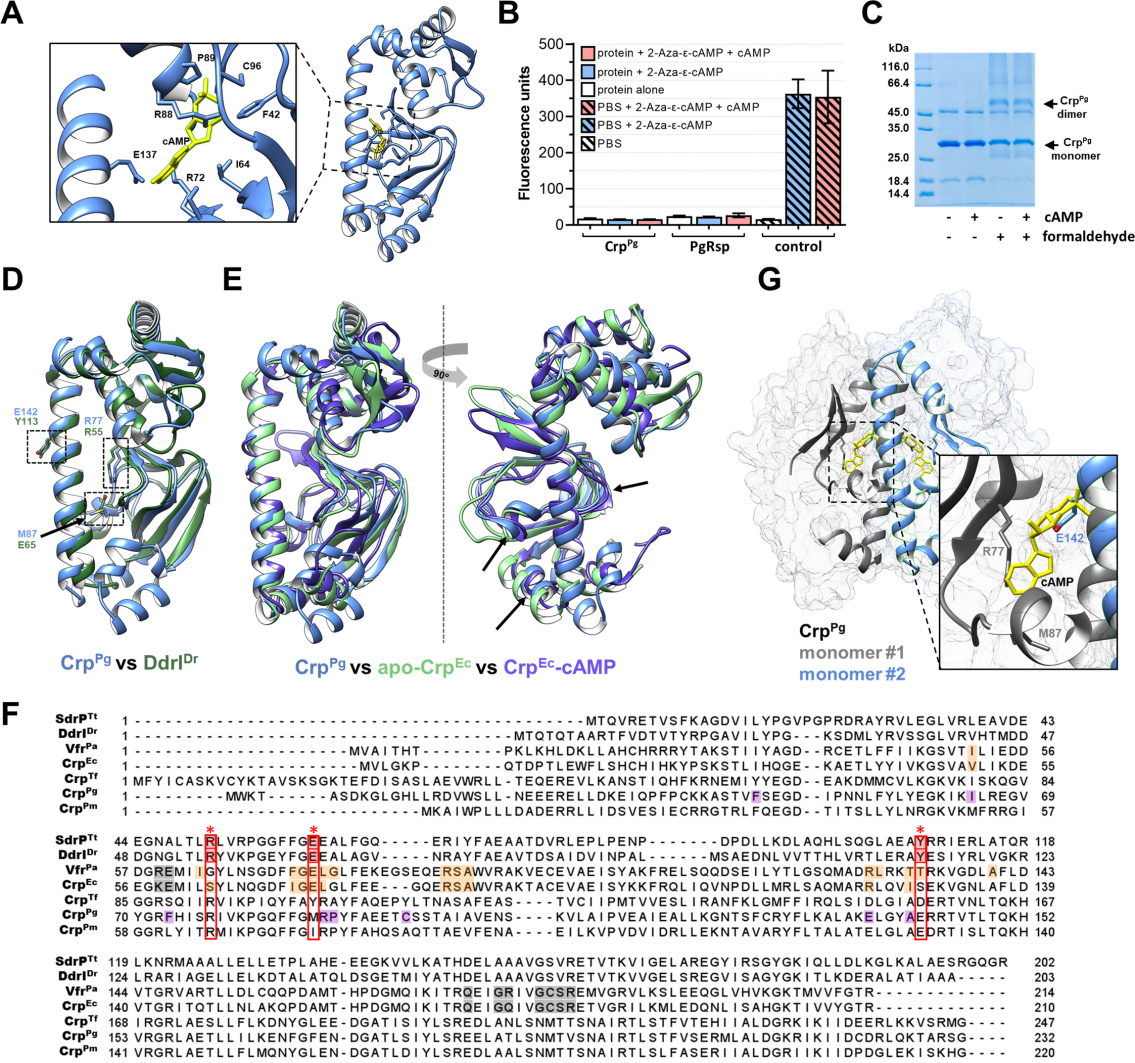
Characterization of Crp^Pg^ protein. **(A)** Potential cAMP-binding pocket with labeled predicted amino acids. Crp^Pg^ structure with cAMP (yellow) was generated using eDock (Zhang et al., 2020). **(B)** Analysis of cAMP binding using its fluorescent analog 2-Aza-ϵ-cAMP. Protein was incubated with 50 µM 2-Aza-ϵ-cAMP (2.5× excess) and with cAMP as a competitor (12.5× excess). After removing the unbound cyclic nucleotide, the fluorescence of the sample was measured. Fluorescence of buffer alone and initial solution of 2-Aza-ϵ-cAMP and 2-Aza-ϵ-cAMP with cAMP were used as controls. Heme-binding PgRsp protein was used as a negative cAMP-binding control. **(C)** SDS-PAGE-based analysis of the cAMP influence on Crp^Pg^ dimer formation using crosslinking with 0.2% formaldehyde. **(D)** Comparison of the three-dimensional structure of Crp^Pg^ (blue) with DdrI^Dr^ (dark green) indicating the amino acids located in the binding pocket. **(E)** Comparison of the three-dimensional structure of the Crp^Pg^ (blue) protein with the Crp^Ec^ protein in the inactive state (apo form; green) and in the active state (Crp^Ec^-cAMP complex; purple) suggests that the Crp^Pg^ protein shows more similarity in the N-terminal domain with the active version of the protein (shown by arrows). **(F)** Amino acid sequence alignment of *P. gingivalis* Crp^Pg^ protein and Crp homologs from *T. forsythia* (Crp^Tf^), *P. macacae* (Crp^Pm^), *E. coli* (Crp^Ec^), *P. aeruginosa* (Vfr^Pa^), *D. radiodurans* (DdrI^Dr^), *T. thermophilus (*SdrP^Tt^). The predicted cAMP-binding amino acids are marked in purple in Crp^Pg^, and experimentally determined cAMP-binding amino acids in other proteins are marked in orange and grey. Amino acids blocking the cAMP-binding pocket are marked in red with an asterisk (*), and homologous amino acids in other Crp homologs are marked with a red frame. **(G)** Analysis of the cAMP-binding pocket and its location in the Crp^Pg^ protein dimer revealed steric hindrance between the glutamic acid (E142) and the cAMP-binding site.

Further, we aimed to find whether Crp^Pg^ functions utilizing the binding of cAMP or other ligands. *In silico* analysis showed that the Crp^Pg^ protein has a potential ligand-binding pocket between the dimerizing α-helix and the N-terminal domain (**Figure 3A**) and the predictions indicated a possibility of cAMP binding (max C-score: 0.6). To verify this, we analyzed the binding of several ligands to the Crp^Pg^ protein. In the initial stage, we examined the binding of cyclic nucleotides or di-nucleotides immobilized on agarose resin. To exclude a physical blockage of the binding site in the protein, we used various ligands labeled in different positions of nucleobases or ribose (**Supplementary Figure S2**). Additionally, we used a cAMP analog with fluorescent properties (2-Aza-ϵ-cAMP) (**Figure 3B**). We observed neither cAMP (**Figure 3B**) nor other ligands (data not shown) binding to the Crp^Pg^ protein. As a control, we used PgRsp, which did not bind 2-Aza-ϵ-cAMP. Moreover, by using EMSA we did not demonstrate the influence of cAMP, cGMP, c-di-AMP, or c-di-GMP on the Crp^Pg^ binding to the promoter sequences (**Supplementary Figure S4C**). Although the Crp^Pg^ protein may exist in the dimeric form, cAMP did not influence its dimerization (**Figure 3C**).

The Crp^Pg^ protein structure is highly similar to the structure of DdrI^Dr^ (**Figures 1C**, **3D**). Moreover, it shows more similarity and structure coverage to the ligand-binding domain of the Crp^Ec^-cAMP complex (protein active form) than to the apo-Crp^Ec^ (not active form) (**Figure 3E**). Predicted cAMP-binding amino acids in the ligand-binding pocket in the Crp^Pg^ differ from those of classical cAMP-binding Crp proteins (**Figures 3A, F**). Our detailed theoretical analyses showed that the Crp^Pg^ protein and other closely related Crp homologs have the conserved arginine in the position relevant to R55 or R51 in DdrI^Dr^ or SdrP^Tt^, respectively (**Figures 3D, F**; **Supplementary Figure S3**). However, in the dimeric state of the Crp^Pg^ protein, the side chain of glutamic acid (E142) located in the position relevant to Y113 in DdrI^Dr^ may block the entrance to the cAMP-binding site located in the second protein monomer (**Figure 3G**).

### 3.4 Crp^Pg^ expression is growth phase-dependent

**Figure 4:**
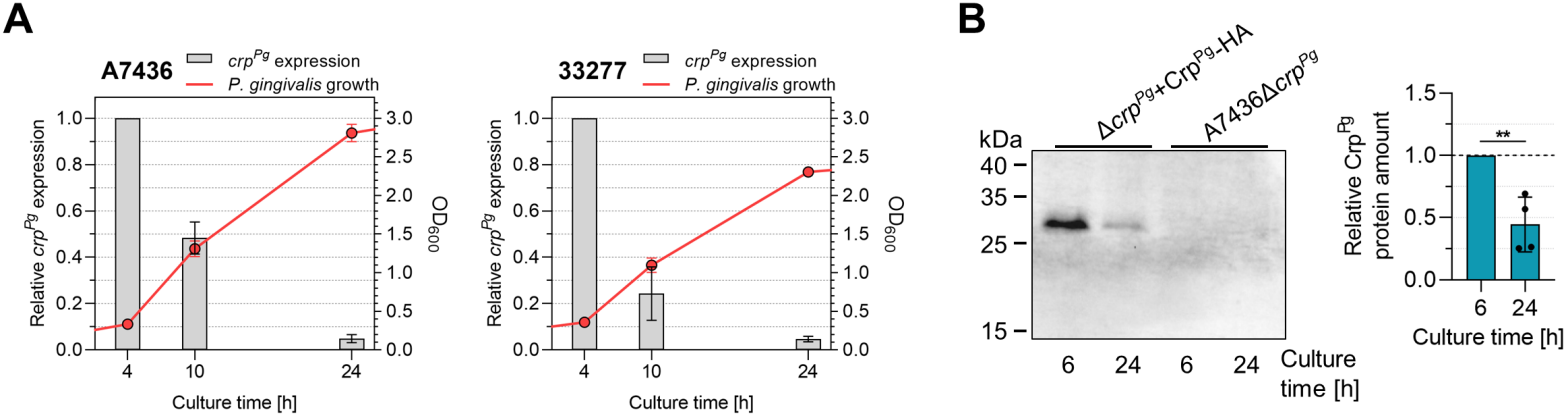
Analysis of *P. gingivalis* Crp^Pg^ expression. **(A)** Influence of the growth phase on the *crp^Pg^* gene expression in A7436 and ATCC 33277 (33277) wild-type strains. Bacteria were grown in iron and heme-replete conditions (Hm) and collected at the indicated time points. The optical density of the culture was determined at 600 nm (OD_600_). Gene expression was examined using RT-qPCR. Transcript levels determined after 10 h and 24 h were shown concerning those determined after 4 h (set as 1). **(B)** The recombinant Crp^Pg^-HA protein production was examined in the Δ*crp^Pg^* +Crp^Pg^-HA complemented strain at the indicated time points using Western blotting with anti-HA antibodies and subsequent densitometric analysis. A7436Δ*crp^Pg^* mutant strain was used as a control. **P<0.01.

Subsequent experiments aimed at further understanding the potential mechanism of the Crp^Pg^ function employed the characterization of the effect of growth conditions on the expression of the *crp^Pg^* gene. First, the *crp^Pg^* transcript level was determined in both A7436 and 33277 wild-type strains. The highest *crp^Pg^* expression was observed in the early growth phase (4 h of the culture), but a significant decrease (up to 6 and 26 times) in *crp^Pg^* transcript levels monitored in the late exponential (10 h of the culture) and early stationary (24 h of the culture) growth phases were detected (**Figure 4A**). Using the Δ*crp^Pg^* +Crp^Pg^-HA complemented strain generated in the A7436 genetic background, in which the Crp^Pg^ protein in fusion with the HA-tag was produced under its native promoter, we confirmed that also Crp^Pg^ protein production is growth phase-dependent. We observed a significant decrease in Crp^Pg^ protein amount (>2 times) in bacteria grown for 24 h compared to analysis carried out in bacteria grown for 6 h (**Figure 4B**), which is in agreement with the transcript levels.

### 3.5 Crp^Pg^ may be important for *P. gingivalis* biofilm formation and interaction with host cells

**Figure 5:**
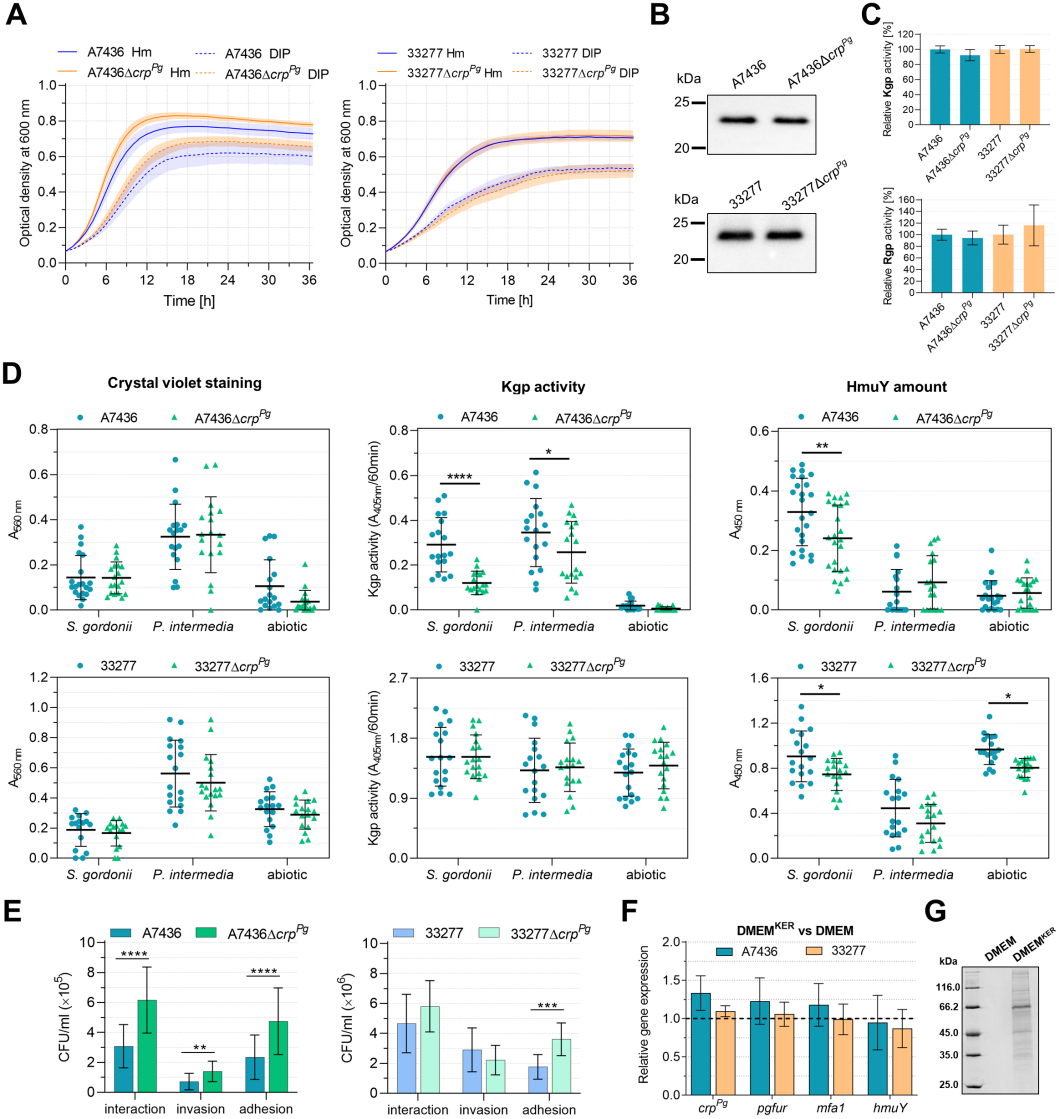
Phenotypic characterization of Δ*crp^Pg^* mutant strains generated in *P. gingivalis* A7436 and ATCC 33277 (33277) wild-type strains. **(A)** The growth of bacteria in liquid culture media containing iron and heme (Hm) or without heme and supplemented with the iron chelator 2,2-dipyridyl (DIP) was monitored over time by measuring the optical density at 600 nm. **(B)** Expression of cell-associated HmuY protein was determined by Western blotting with anti-HmuY antibodies. **(C)** The relative gingipain activities of the whole *P. gingivalis* cultures were measured using lysine-specific (Kgp) and arginine-specific (Rgp) substrates. The activity of the wild-type strains was set as 100%. **(D)** Biofilm formation of *P. gingivalis* on an abiotic surface and *S. gordonii* or *P. intermedia* pre-coated plates. Biofilms formed were determined using crystal violet staining, concerning the Kgp activity of whole bacterial cells embedded in biofilm structures (Kgp activity) or the production of HmuY protein by whole bacterial cells forming the surface of biofilm structures (HmuY amount). The biofilm formation assay was performed 4 independent times, every time with 2 biological replicates for *S. gordonii* and *P. intermedia* biofilm, and 3 biological replicates for *P. gingivalis*. **(E)** The interaction of *P. gingivalis* with host cells. The ability to invade and adhere to host cells was analyzed using a *P. gingivalis*-gingival keratinocytes co-culture model. The number of viable bacteria was shown as colony-forming units per ml (CFU/ml). Adhesion – live bacteria attached to keratinocytes; invasion – live bacteria that invaded keratinocytes; interaction – the total number of live bacteria that invaded and adhered to keratinocytes. **(F)** Gene expression was determined in *P. gingivalis* cultured for 4 h in the medium collected after 24-h keratinocytes culture (DMEM^KER^) in comparison to the fresh medium (DMEM), the latter set as 1. Gene expression was examined using RT-qPCR. **(G)** Protein pattern of fresh DMEM and DMEM^KER^ determined with SDS-PAGE. Results are shown as mean ± SE **(A)** or mean ± SD **(C-F)**. *P<0.01, **P<0.01, ***P<0.001, ****P<0.0001.

Following analysis of the potential regulation of the expression of *P. gingivalis* virulence factors by the Crp^Pg^, we examined *P. gingivalis* growth alone or with other bacteria or host cells. First, we examined how *crp^Pg^* deletion affects the phenotype of *P. gingivalis*, with a focus on the effect on its virulence potential. The inactivation of the *crp^Pg^* gene did not influence the 33277Δ*crp^Pg^* mutant strain proliferation (**Figure 5A**). The A7436Δ*crp^Pg^* mutant strain grew slightly better than the A7436 wild-type strain, regardless of the growth conditions, but the differences were not statistically significant (**Figure 5A**). Its wild-type phenotype was restored when the Crp^Pg^-HA protein was produced in the A7436Δ*crp^Pg^* mutant strain (**Supplementary Figure S5A**). The analysis of the production and activity of the two groups of the most important virulence factors, namely hemophore-like HmuY protein and gingipains (Kgp and Rgp) showed no differences between the wild-type and mutant strains (**Figures 5B, C**).

Since Crp^Pg^ regulates *mfa* operon, we analyzed the influence of the *crp^Pg^* gene on the ability of the bacterium to form biofilm. *P. gingivalis* mono-cultures were formed directly on an abiotic surface or on an abiotic surface first colonized by *S. gordonii* or *P. intermedia*. Using this simplified model, we aimed to examine the influence of the lack of the *crp^Pg^* gene on *P. gingivalis* adherence and biofilm formation on an abiotic surface. To mimic the environment of the oral cavity, we employed plates pre-coated with *S. gordonii* or *P. intermedia.* Both species colonize the oral cavity earlier than *P. gingivalis*, form biofilms on an abiotic surface better than *P. gingivalis* alone, and play a crucial role in recruiting *P. gingivalis* into the biofilm. In addition, these bacteria were selected due to their proven interactions. *P. gingivalis* fimbral protein Mfa1 interacts with the *S. gordonii* SspA/B proteins, while *P. gingivalis* fimbral protein FimA binds to *S. gordonii* GAPDH (Kuboniwa and Lamont, 2010). In the interaction between *P. gingivalis* and *P. intermedia*, a crucial role is played by *P. gingivalis* gingipains (Kamaguchi et al., 2001). Biofilm formation was first determined using crystal violet staining (**Figure 5D**, left panel). Although the inactivation of the *crp^Pg^* gene resulted in a lower tendency to form biofilm structures on an abiotic surface in the case of both strains, especially the A7436 strain, the differences were not statistically significant. Biofilm formation was further examined in correlation to selected *P. gingivalis* surface proteins. Since no change in HmuY or Kgp production or activity, respectively, was demonstrated between wild-type and mutant strains, and both proteins function in part in cell-associated forms, the *P. gingivalis* biofilm formation was also determined using specific anti-HmuY antibodies or enzymatic reaction with a specific substrate for Kgp. When the biofilm was examined on the abiotic surface colonized first by *S. gordonii* or *P. intermedia* and related to the Kgp activity, only the Δ*crp^Pg^* mutant strain constructed in the A7436 strain exhibited a lower ability to form biofilm structures (**Figure 5D**, middle panel). When the formation of biofilm was correlated with the production of HmuY protein, inactivation of the *crp^Pg^* gene in both A7436 and 33277 strains resulted in a lower ability to form biofilm structures when *P. gingivalis* was incubated on plates colonized first by *S. gordonii* (**Figure 5D**, right panel). This effect was also observed in the case of the Δ*crp^Pg^* mutant strain constructed in the 33277 strain, grown on an abiotic surface.

The ability to interact with host cells (adhere to and invade host cells) was analyzed using a *P. gingivalis*-gingival keratinocytes co-culture model (Śmiga et al., 2023). These abilities increased in the case of the Δ*crp^Pg^* mutant strain constructed in the A7436 genetic background (**Figure 5E**). The observed changes were eliminated when the Δ*crp^Pg^* +Crp^Pg^-HA complemented strain was examined (**Supplementary Figure S5B**). In the case of the Δ*crp^Pg^* mutant strain constructed in the 33277 genetic background, although we detected a slightly increased interaction of this strain with the host cells, the difference was not statistically significant. However, we observed a higher ability of the 33277Δ*crp^Pg^* strain to adhere to keratinocytes, similarly as in the case of the A7436Δ*crp^Pg^* strain (**Figure 5E**). We also examined the expression of selected genes in bacteria grown for 4 h in the culture medium collected from 24-hour gingival keratinocyte cultures (DMEM^KER^). Slightly increased *crp^Pg^*, *pgfur*, and *mfa1* gene expressions were observed when the A7436 strain was grown in DMEM^KER^, whereas no changes were found in the case of the 33277 strain (**Figure 5F**). These results indicated that the external environment of host cells, which is enriched with proteins secreted by keratinocytes (**Figure 5G**) can induce *crp^Pg^*-dependent gene expression.

### 3.6 Inactivation of the *crp^Pg^* gene may alter glycosylation

**Figure 6:**
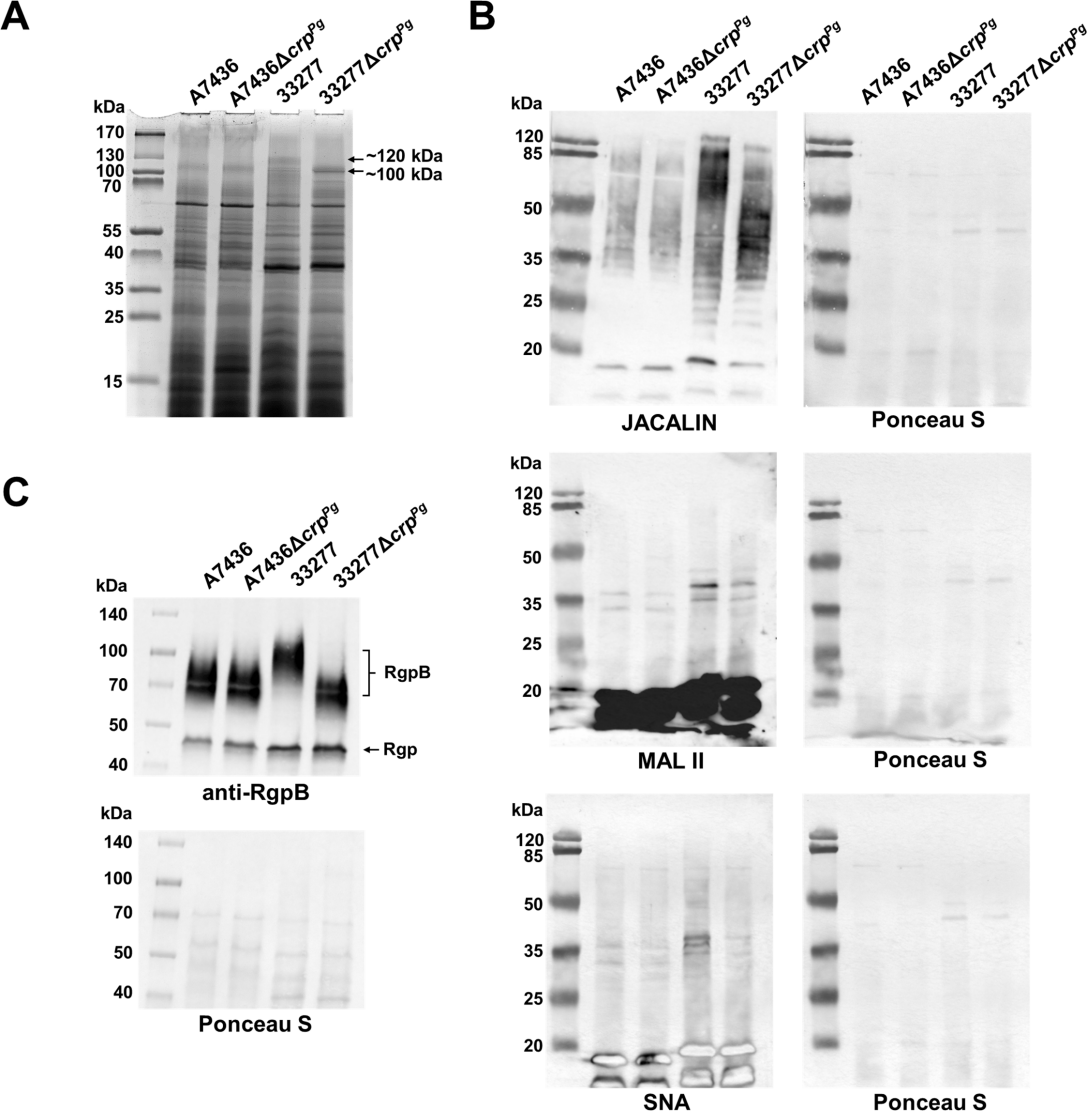
Analysis of Crp^Pg^ influence on *P. gingivalis* glycosylation. **(A)** Protein production was examined in whole *P. gingivalis* wild-type and Δ*crp^Pg^* mutant cell lysates by SDS-PAGE. After electrophoresis, proteins were visualized using CBB G-250. The arrows indicate ~100 and ~120 kDa protein bands in 33277 and 33277Δ*crp^Pg^* which were analyzed with MS. **(B)** Glycosylation patterns were analyzed using lectin blotting with JACALIN (specific to 3-substituted GalNAcα, and tolerates substitutions at the position 3 of GalNAc *via* either α or β linkage, including GalNAc, GlcAc, Gal, and longer oligosaccharides), SNA (specific to sialic acid linked to the terminal galactose with α-2,6 or α-2,3 linkage), and MAL II (specific to α2-3-sialylated Galβ1-3GalNAc in *O*-glycans and tolerates substitutions at the 6-position of GalNAc; e.g., sialylation, sulfation, GlcNAc) lectins (Bojar et al., 2022). **(C)** Analysis of cell-associated RgpB protein production was examined by Western blotting with anti-RgpB antibodies. RgpB – RgpB protein modified with LPS, Rgp – catalytic domains of RgpA and RgpB. Protein loading after the transfer was verified by Ponceau S staining.

In the next step of characterizing the Crp^Pg^ role in *P. gingivalis*, proteins present in whole bacterial cell lysates were analyzed by SDS-PAGE. The main differences were visible in the disappearance of the ~120-kDa band with a simultaneous intensification of the ~100-kDa band in the 33277Δ*crp^Pg^* mutant strain compared to the 33277 wild-type strain (**Figure 6A**, **Supplementary Figure S6**). In the A7436 and A7436Δ*crp^Pg^* strains, only ~100-kDa band was visible (**Figure 6A**, **Supplementary Figure S6**). Using MS we confirmed that a protein ascribed to ~100-kDa and ~120-kDa bands is a putative zinc carboxypeptidase (91.5 kDa) encoded by the *PGA7_RS01035* (*PGN_0335*/*PG0232*) gene (**Supplementary Figure S6**). This led us to conclude that the difference in the protein molecular mass may result from posttranslational modification, such as glycosylation. However, the search for glycosylated peptides after MS analysis was inconclusive. Reactivity with JACALIN, SNA, and MAL II lectins confirmed previously shown (Śmiga et al., 2024a) differences in glycosylation patterns between A7436 and 33277 strains (**Figure 6B**). Moreover, it showed that the deletion of the *crp^Pg^* gene caused a change in the glycosylation pattern, mainly in the 33277 strain. Among *P. gingivalis* proteins modified by glycosylation is RgpB. To detect changes in RgpB glycosylation in the Δ*crp^Pg^* mutant strains, we used anti-RgpB antibodies and determined the mass of produced RgpB. Similarly to the putative zinc carboxypeptidase, also in the 33277Δ*crp^Pg^* mutant strain, RgpB had a lower mass than in the 33277 wild-type strain. Moreover, the observed RgpB mass corresponded to the mass of the protein produced in the A7436 wild-type and A7436Δ*crp^Pg^* mutant strains (**Figure 6C**). Changes in the presented phenotype can partially be explained by the down-regulation of *PG1135* (*wbaQ*, sugar transferase/phosphoglycosyltransferase), *PG0119* (WecB/TagA/CpsF family glycosyltransferase), and up-regulation of *PG0043* (family 20 glycosylhydrolase/beta-hexosaminidase) genes (**Supplementary Table S3**), all involved in glycosylation and sugar metabolism.

### 3.7 Crp^Pg^ may influence energy metabolism

Results from phenotypic and transcriptomic analyses suggested that Crp^Pg^ may regulate processes dependent on energy production. Indeed, in our transcriptomic analysis, we observed changes in the expression of genes involved in energy metabolism. The best example is a system composed of genes encoding V-type ATP synthase subunits (*PG1803-PG1807* operon, encoding AtpD, AtpI, and AtpB) whose expression increased in the A7436Δ*crp^Pg^* mutant strain (**Supplementary Table S3**). Another example is a system composed of FtsX (FtsX-like permease family protein and two ABC transporter permeases) and FtsE (ABC transporter ATP-binding protein) proteins encoded by *PG0682-PG0685* whose expression increased in the mutant strain (**Supplementary Table S3**). Classical Crp proteins play a key role in regulating energy metabolism, a function often reflected in the levels of various signal molecules, including cAMP and c-di-AMP, as well as in the ratio of other critical nucleotides, such as ATP/ADP and NAD^+^/NADH. Therefore, we determined ATP, ADP, NAD^+^, and NADH contents (**Supplementary Figures S7A, B**). While NAD^+^ and NADH amounts did not change in both Δ*crp^Pg^* mutant strains, the amount of ATP and ADP pull decreased in the A7436Δ*crp^Pg^* mutant strain after 6 h of the culture (~20% decrease), and in the 33277Δ*crp^Pg^* mutant strain after culture for 24 h (~29%). Moreover, the decrease was up to 20% or 70% for ATP or ADP amounts, respectively, in the 33277Δ*crp^Pg^* mutant strain, whereas no changes were found in the case of the A7436Δ*crp^Pg^* mutant strain.

Additionally, we determined PDE activity to demonstrate whether the lack of the functional *crp^Pg^* gene affects the metabolism of cyclic nucleotides; however, no differences between the wild-type and corresponding mutant strains were observed (**Supplementary Figure S7C**). This is in agreement with the RNA-seq analysis where no change in gene expression whose products are involved in cyclic nucleotide metabolism was found.

## 4 Discussion

Bacteria, especially host-associated pathogens, must respond quickly to internal and external environmental changes through cell-to-cell contact or detection of signals. Such responses are necessary to adapt their metabolic pathways to transit from planktonic to biofilm conditions and to enhance viability and pathogenicity in the host. Among many signals, bacteria use cyclic nucleotides and di-nucleotides, molecules involved in intracellular and extracellular signaling (Liu et al., 2024). In *P. gingivalis*, c-di-AMP is assumed to be the primary signaling molecule involved in controlling growth, biofilm formation, cell envelope composition, interaction with other bacteria, and virulence (Chaudhuri et al., 2014; Moradali et al., 2022; Ghods et al., 2024). Therefore, this study examined the Crp^Pg^ protein, ascribed to the CRP/FNR superfamily, as a potential regulator responding to cyclic nucleotide or di-nucleotide levels.

It has been well-documented that many transcription factors belonging to the CRP/FNR superfamily regulate virulence-associated genes, including those involved in biofilm formation (Soberon-Chavez et al., 2017; Korner et al., 2003). Some examples include *Listeria monocytogenes* PrfA (Stelling et al., 2010), *M. tuberculosis* Cmr (Smith et al., 2017), *M. tuberculosis* Crp^Mt^ (Akhter et al., 2007, 2008), *Staphylococcus aureus* ArcR (Fu et al., 2023), *Vibrio cholerae* Crp^Vc^ (Liang et al., 2007; Fong and Yildiz, 2008), *P. aeruginosa* Vfr^Pa^ (Albus et al., 1997; Beatson et al., 2002; Fuchs et al., 2010), and *Xanthomonas campestris* CLP^Xa^ (Chin et al., 2010). In many bacteria, this feature is controlled by Crp proteins due to the regulation of the expression of fimbrial proteins engaged in fimbriae formation (Tsai et al., 2017; Lin et al., 2016; Park et al., 2006; Hasegawa and Nagano, 2021). This was one of the reasons we aimed to explore the importance of the Crp^Pg^ for *P. gingivalis* biofilm formation, being crucial for its pathogenicity. Although in the *P. gingivalis* Δ*crp^Pg^* mutant strain the expression of *mfa* genes (encoding structural and accessory fimbrial proteins) (Ikai et al., 2015; Hall et al., 2017), was higher, analysis of mono-species *P. gingivalis* biofilm on an abiotic surface or biofilm composed of *P. gingivalis* and *S. gordonii* or *P. gingivalis* and *P. intermedia* revealed a lower capacity of the mutant strain to form biofilm structures. It seems that the Crp^Pg^ may down-regulate directly or indirectly the expression of *mfa* genes during *in vivo* biofilm formation and regulate other proteins engaged in this process to preserve the dynamic life of the biofilm consortium. Therefore, we postulate the engagement of the Crp^Pg^ in regulating bacterial cross-talk during the transition from planktonic to biofilm-embedded bacteria. This effect was better visible in the case of the more virulent A7436 strain. The expression of two *P. gingivalis* regulators belonging to the CRP/FNR superfamily, namely Crp^Pg^ (examined in this study) and HcpR (Belvin et al., 2019b), was down-regulated in the bacterial consortium containing the W83 strain but up-regulated in the consortium comprising the 33277 strain (Zhang et al., 2019), which may support differences observed between strains examined in our study.

The glycosylation of bacterial surface molecules is the other aspect that may influence biofilm formation. As demonstrated in this study on the RgpB example and the general glycosylation pattern of cell lysate components, deletion of the *crp^Pg^* gene affects this process. This may prove that Crp^Pg^ regulates glycosylation, which differs between both analyzed strains. Differences in glycosylation between strains may result from different expressions of glycosyltransferases in strain 33277 which does not produce the capsule and the lack in the A7436 strain two glycosyltransferases encoded in the 33277 strain by *PGN_0225* and *PGN_0227* genes (Shoji et al., 2018). Therefore, it can be assumed that the influence of the *crp^Pg^* deletion may affect analyzed strains differently due to the different glycosylation processes.

Further, we attempted to analyze the significance of the Crp^Pg^ for *P. gingivalis* interaction with host cells, another aspect important for pathogenicity. Surprisingly, the inactivation of the *crp^Pg^* gene resulted in a higher ability of the mutant cells to adhere to and invade keratinocytes. Therefore, we suggest that *in vivo* Crp^Pg^ may modulate the expression of genes whose products allow a lower intracellular *P. gingivalis* replication. This mechanism may lead to a balanced interaction that ensures lower adhesion and intracellular bacterial replication while preventing excessive damage to the host. Such a mechanism may also influence the evasion of the host’s immune response. As shown by others, genes down-regulated in *P. gingivalis* inoculated to the rat oral cavity, which enabled contact with and invasion of bacteria into gingival cells, included *PGA7_RS08955* (*PG2008*) gene (encoding TonB-dependent receptor), *PGA7_RS05990* (*PG0684*), *PGA7_RS05995* (*PG0683*) and *PGA7_RS06000* (*PG0682*) genes (encoding FtsX and FtsE proteins), and *PGA7_RS08820* (*PG1975*) (encoding HagC hemagglutinin) (Zhao et al., 2015). Importantly, these genes were up-regulated in the Δ*crp^Pg^* mutant strain. Others found that the production of the Crp^Pg^ protein was increased when *P. gingivalis* was cultured in the cell-free medium collected from gingival epithelial cell cultures (Zhang et al., 2005). In agreement with this study, we found that the transcript encoding Crp^Pg^ was produced at higher levels when bacteria were cultured in the cell-free medium collected from gingival keratinocyte cultures. However, this effect was observed only in the case of the A7436 strain. Therefore, we suspect that the Crp^Pg^ may sense and respond to so far unknown signaling molecules or proteins produced by host cells in a strain-specific manner, being more active in more virulent strains.

Bacterial transcription factors often form a multi-layer regulatory network which is crucial for efficient virulence. Previous reports demonstrated that overexpression of the extracytoplasmic function sigma factor PG0162 in *P. gingivalis* W83 strain resulted in up-regulated expression of the *crp^Pg^* gene (Dou et al., 2016). This points to the assumption that Crp^Pg^ could also regulate other genes involved in pathogenicity since the PG0162 protein regulates the expression of genes engaged in *P. gingivalis* virulence, including genes encoding gingipains (Dou et al., 2016). However, we did not detect differences in gingipains’ activity between the wild-type and mutant strains, although the expression of the *rgpA* gene was increased in the Δ*crp^Pg^* mutant strain. Previously, we found lower expression, mainly in the early stationary growth phase, of *crp^Pg^* and *pgrsp* genes encoded in the mutant strains lacking the functional *pgfur* (constructed in both A7436 and 33277 strains) (Śmiga et al., 2019b). Therefore, our findings add the *crp^Pg^* gene to the *P. gingivalis* multi-layer regulatory network. This assumption might also be supported by the observation that the Δ*crp^Pg^* mutant strain was significantly affected by the expression of genes encoding several transcription factors.

Based on the gene expression analysis it seems that Crp^Pg^ may also be important for the maintenance of proper redox conditions required for effective cellular metabolism. The intracellular environment is maintained in a reduced condition due to the functioning of proteins containing redox-active cysteine residues, including the thioredoxin system composed of thioredoxin reductase (TrxR), the corresponding thioredoxin substrate, and the cofactor, NADP^+^/NADPH (Zeller and Klug, 2006; Lu and Holmgren, 2014). This system is used in several cellular processes, including transcription, DNA replication and repair, cell growth, and division. Thioredoxins not only participate in reducing cytoplasmic proteins but can also directly reduce hydrogen peroxide (Zeller and Klug, 2006), which can be produced by *S. gordonii* (Ślęzak et al., 2020). *P. gingivalis* genome contains genes encoding thioredoxin reductase (TrxR encoded by a *trxB* gene), thioredoxin (TrxA encoded by a *trxA* gene), and a few thioredoxin family proteins. Although in the Δ*crp^Pg^* mutant strain, lower levels of mRNA encoding TrxA were determined, no difference in the expression of TrxB was demonstrated. In addition, thioredoxins may contribute to SoxR (a component of the SoxRS regulon which is engaged in the response to superoxide in enteric bacteria) regulation by affecting the disassembly and reassembly of the [2Fe-2S] clusters (Zeller and Klug, 2006). In the Δ*crp^Pg^* mutant strain, the expression of the gene encoding a SoxR homolog and 4Fe-4S- binding proteins was decreased. Other examples are short-chain dehydrogenases/reductases (SDR) and NADP^+^/NADPH-dependent oxidoreductases, which serve as a redox sensor system important in cell metabolism, transcription, and signaling (Kavanagh et al., 2008). In the Δ*crp^Pg^* mutant strain, the expression of the SDR family oxidoreductase encoding gene (*PGA7_RS09250*, *PG2069*) was significantly decreased. In contrast, another NAD(P)-dependent oxidoreductase, encoded by the *PGA7_RS02260* (*PG1504*) gene, was up-regulated (1.77 fold change) but the difference was not statistically significant. Also, no changes in gene expression in the Δ*crp^Pg^* mutant strain were found in the case of many other oxidoreductases. Therefore, we determined the intracellular contents of NAD^+^, NADH, ATP, and ADP, whose ratios may be crucial in maintaining metabolic status and bacterial cell survival. Analysis of NAD^+^ and NADH contents demonstrated no differences between the wild-type and mutant strains. In contrast, a lower ATP and ADP pool was found but at different times of growth curves in both examined strains, with lower contents of respective nucleotides in the 33277Δ*crp^Pg^* mutant strain. This data suggests the engagement of the Crp^Pg^ in energy metabolism, which is also a strain-specific feature.

Results gained from our experiments demonstrated that the Crp^Pg^ is one of the regulatory factors participating in *P. gingivalis* survival. However, based on the results obtained, we are not able to identify the stimulus to which the protein responds or ligand bound to this protein in the process of gene expression regulation. Classical Crp protein acts as a repressor or activator of transcription. As an activator, it binds to DNA and interacts with RNA polymerase (RNAP), which results in transcription, and as a repressor, it binds to the RNAP-binding site directly or after the recruitment of a co-repressor, which prevents transcription. The theoretically predicted function of the Crp^Pg^ is connected with binding cyclic nucleotides or di-nucleotides and nucleic acids. However, we have not demonstrated the sensing or binding of such ligands. We showed that when added to the bacterial cultures or the purified protein sample, those ligands did not affect *P. gingivalis* growth, did not bind to the protein, did not influence dimer formation, and did not influence DNA-binding ability. This is not surprising because such proteins belonging to the CRP/FNR family are already known. Some atypical Crp proteins, such as DdrI^Dr^ (DNA damage response regulator) from *D. radiodurans* and SdrP^Tt^ (stationary phase-dependent regulatory protein/oxidative stress-responsive activator) from *T. thermophilus* also do not bind cAMP. The activation of these proteins occurs independently of added effector molecules *in vitro* (Agari et al., 2008, 2012; Wang et al., 2024). Importantly, their three-dimensional structures (Agari et al., 2008, 2012; Wang et al., 2024) resemble that of the DNA-bound form of *E. coli* Crp^Ec^ complexed with cAMP and DNA (Passner and Steitz, 1997). In their case, the cAMP-binding pocket is blocked by the side chains of arginine, glutamic acid, and tyrosine, for example by R55, E65, and Y113 in DdrI^Dr^ (Agari et al., 2008, 2012; Wang et al., 2024). However, compared to those proteins, the active form of Crp^Pg^ is caused by a steric hindrance of another amino acid. In the dimeric form, the side chain of E142 located in the position relevant to Y113 in DdrI^Dr^ may block the ligand entrance to the cAMP-binding site located in the second protein monomer. Another Crp homolog, which can belong to this subfamily is Crp^Gv^ from *Gardnerella vaginalis* (Dong et al., 2024). In this protein, the first 23 amino acids block the ligand-binding pocket, which prevents cAMP binding. The authors suggested that the Crp^Gv^ regulates gene expression through ligand-independent conformational change caused by interaction with a protein partner, leading to DNA or RNAP binding.

It seems that also Crp^Pg^ may not require an allosteric effector molecule to bind DNA or may bind another cofactor or protein partner. One such mechanism uses SdrP^Tt^ from *T. thermophilus* whose activity is based on the changing protein concentration (Agari et al., 2010). Based on the differential expression of the *crp^Pg^* gene, a similar mechanism could be used by the Crp^Pg^ protein. The phenotypic characterization of the growth under laboratory conditions suggested that the Crp^Pg^ may not be important in the later growth phases when *P. gingivalis* density is higher but the availability of nutrients is lower. Indeed, the expression of the *crp^Pg^* gene significantly decreased in the late exponential growth phase, at both the transcript and protein levels, and almost disappeared in the stationary growth phase. Indeed, the expression of the *crp^Pg^* gene significantly decreased in the late exponential growth phase, at both the transcript and protein levels, and almost disappeared in the stationary growth phase, which is in contrast to other *P. gingivalis* transcription regulators, such as PgFur and PgRsp, whose expression does not depend on the growth phase (Śmiga et al., 2019b; Śmiga and Olczak, 2019). In contrast, expression of the homologous *T. thermophilus sdrP^Tt^* gene increased significantly upon entry into the stationary growth phase (Agari et al., 2008). This may explain the observation that the inactivation of the *crp^Pg^* gene did not result in growth retardation, which is in contrast to the inactivation of the homologous *sdrP^Tt^* and *ddrI^Dr^* genes, both required in later growth phases (Agari et al., 2008; Yang et al., 2016; Meyer et al., 2018). Based on the varying *crp^Pg^* gene expression in different growth phases, with the highest transcript and protein levels in the early growth phase, we assumed that Crp^Pg^ is required for *P. gingivalis* growth when bacterial density is low and rapidly replicating bacteria exhibit a highly active metabolism. This suggests that Crp proteins may function differentially depending on the environmental challenges and bacterial lifestyle. It is worth mentioning here that the lack of the functional *crp^Pg^* gene resulted in significantly decreased expression of the UstA protein. This protein is used to improve *P. gingivalis* growth in the stationary growth phase, especially during stress conditions, such as exposure to oxygen (Kikuchi et al., 2005). However, based on similar growth patterns of the wild-type and Δ*crp^Pg^* mutant strains, it seems that neither during the early exponential growth phase nor in the stationary growth phase *P. gingivalis* may not require UstA protein when grown in liquid culture media. A similar finding was reported for stationary phase survival protein E (SurE) of *Burkholderia pseudomalleli*, which was more important for *in vivo* bacterial growth (Techawiwattanaboon et al., 2015).

Proteins ascribed to the CRP/FNR subfamily, including the Crp family are highly heterogeneous. Based on the size of amino acid residues located in the cAMP-binding site or other modifications of the protein structure, the ligand they bind, or the lack of ligand binding ability, proteins belonging to the Crp family may belong to two Crp subfamilies: 1) whose members are effector-dependent regulators and 2) whose members are effector-independent regulators. We assume that the Crp^Pg^ belongs to the second Crp subfamily.

The findings presented in this report suggest that the Crp^Pg^ can differentially sense and respond to signals produced by other bacteria and host cells. It can sense other stimuli compared to its homologs, such as SdrP^Tt^ or DdrI^Dr^, which could result from different environmental challenges. The expression of the *crp^Pg^* gene changes over time when bacteria are grown in liquid culture media, with expression changes following increasing cell density and metabolic changes, with the highest mRNA and protein levels observed in the early exponential growth phase. Therefore, changes in the expression of the *crp^Pg^* gene can be correlated with *in vivo* status. In an early stage of periodontitis, characterized by a lower number of bacteria and higher metabolism to ensure cell replication and a higher tendency of bacteria to initiate biofilm formation, the Crp^Pg^ is produced at higher levels. However, in an advanced disease stage, characterized by the proximity of bacterial cells and more dynamic biofilm structures, the protein is expressed at lower levels. We assume that the Crp^Pg^ may act as a repressor that binds to the RNAP-binding site directly or after the recruitment of a not-identified partner. This could allow the participation of Crp^Pg^ in *P. gingivalis* pathogenicity due to the engagement in the regulation of the transition from planktonic to biofilm bacterial lifestyle. Moreover, modulated *crp^Pg^* gene expression can be important for the precise regulation of genes encoding virulence factors required to invade host cells and regulate bacterial replication inside host cells to evade the host’s immune response.

## Data Availability

The data underlying this article are available in the article and its on-line supplementary material. RNA-seq data are available from the Gene Expression Omnibus (GEO; NCBI) and can be accessed with GSE281934 and GSE275439. The mass spectrometry proteomics data have been deposited in the ProteomeXchange Consortium via the PRIDE (Perez-Riverol et al. 2022) partner repository with the dataset identifier PXD057991 and 10.6019/PXD057991.
