# Supplementary material for: cAMP-independent Crp homolog adds to the multi-layer regulatory network in Porphyromonas gingivalis
Source: Front Cell Infect Microbiol. 2025 Apr 16;15:1535009. doi: 10.3389/fcimb.2025.1535009 (PMC12040651; doi:10.3389/fcimb.2025.1535009)
Supplement: Supplementary file 1 [file DataSheet1.pdf]

Supplementary data for:

cAMP-independent Crp homolog adds to the multi-layer regulatory network in *Porphyromonas gingivalis*

Michał Śmiga[1]*, Ewa Roszkiewicz[1], Paulina Ślęzak[1], Michał Tracz[2], Teresa Olczak[1]

[1]Laboratory of Medical Biology, Faculty of Biotechnology, University of Wrocław, 14A F. Joliot-Curie St., 50-383 Wrocław, Poland

[2]Laboratory of Protein Mass Spectrometry, Faculty of Biotechnology, University of Wrocław, 14A F. Joliot-Curie St., 50-383 Wrocław, Poland

*Correspondence: Michał Śmiga, michal.smiga@uwr.edu.pl

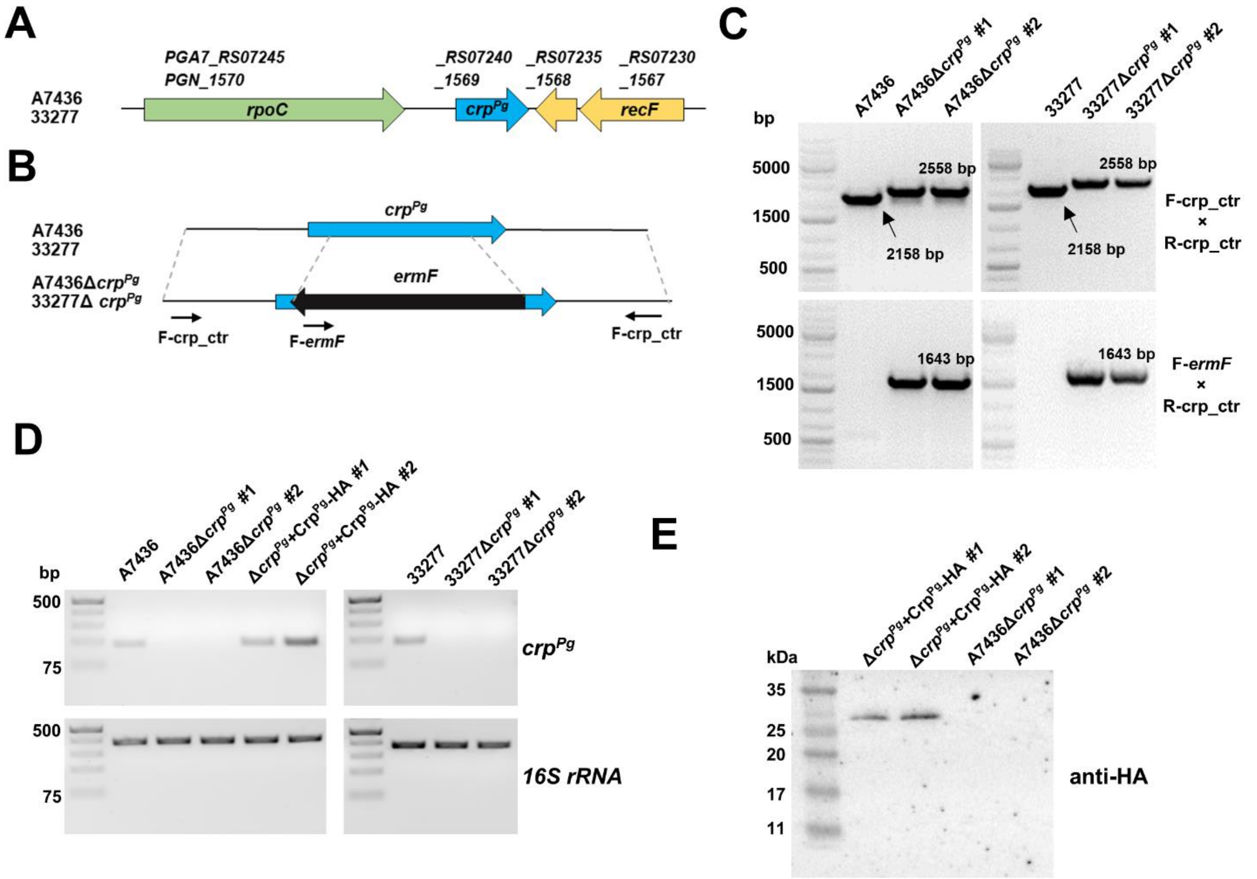

Fig. S1. Confirmation of the construction of modified *P. gingivalis* strains. (A) Schematic presentation of the genome fragment containing the *crp*^{Pg} gene and its flanking regions. (B) Construction of *P. gingivalis* Δ*crp*^{Pg} mutant strains. Validation of the mutation introduced by deleting most of the gene and introducing the *ermF* antibiotic resistance cassette was performed using PCR (C) and indicated primers (Table S2). (D) The expression of the *crp*^{Pg} gene in Δ*crp*^{Pg} mutant and Δ*crp*^{Pg}+Crp^{Pg}-HA complemented strains was confirmed by RT-PCR and primers used for the qPCR (Table S2). The *16S rRNA* gene was used as a control. (E) Crp^{Pg} protein production in the complemented Δ*crp*^{Pg}+Crp^{Pg}-HA strain was confirmed using Western blotting with anti-HA antibodies, and the A7436Δ*crp*^{Pg} mutant strain was used as a control.

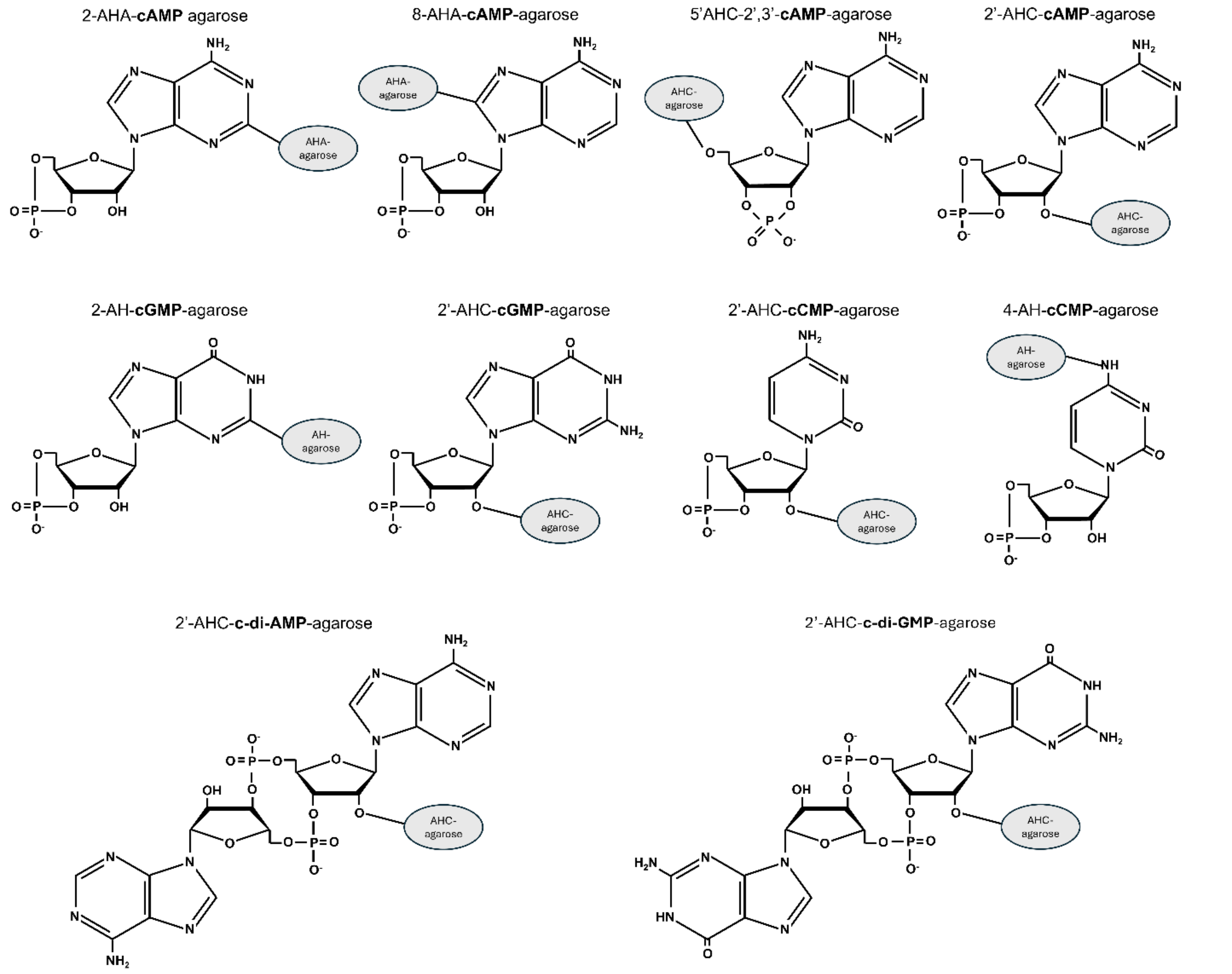

Fig. S2. Schematic presentation of immobilized ligands on resins used in affinity chromatography to determine the binding of cAMP or other cyclic nucleotides by Crp^{Pg}. Cyclic nucleotides were immobilized on agarose gels with AHA – aminohexylamine, AHC – aminohexylcarbamoyl, or AH – aminohexyl linkers. All resins were purchased from the Biolog Life Science Institute.

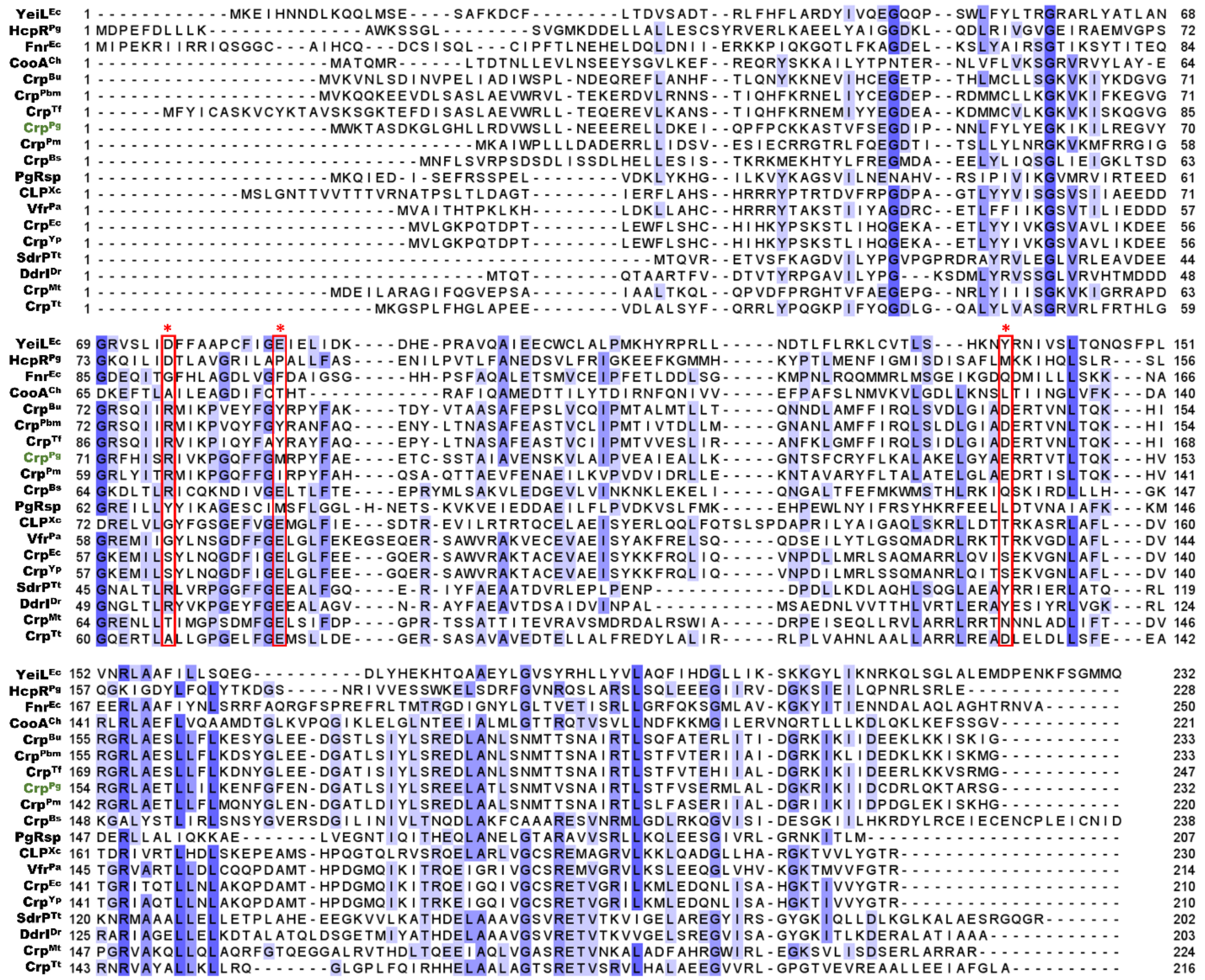

Fig. S3. Amino acid sequence alignment of *P. gingivalis* Crp^{Pg} protein (green) and selected CRP/FNR superfamily homologs (black) from *E. coli* (Crp^{Ec}, YeiL^{Ec}, Fnr^{Ec}), *P. gingivalis* (HcpR^{Pg}, PgRsp), *Tannerella forsythia* (Crp^{Tf}), *Carboxydothermus hydrogenoformans* (CooA^{Ch}), *Bacteroides uniformis* (Crp^{Bu}), *Parabacteroides merdae* (Crp^{Pbm}), *Porphyromonas macacae* (Crp^{Pm}), *Xanthomonas campestris* (CLP^{Xc}), *Pseudomonas aeruginosa* (Vfr^{Pa}), *Yersinia pestis* (Crp^{Yp}), *Bacillus subtilis* (Crp^{Bs}), *Mycobacterium tuberculosis* (Crp^{Mt}), *Thermus thermophilus* (Crp^{Tt}, SdrP^{Tt}), *Deinococcus radiodurans* (DdrI^{Dr}). Shades of blue indicate the similarity between amino acid sequences. An asterisk and a red frame indicate the location of DdrI^{Dr} homologous amino acid residues blocking the cAMP-binding pocket.

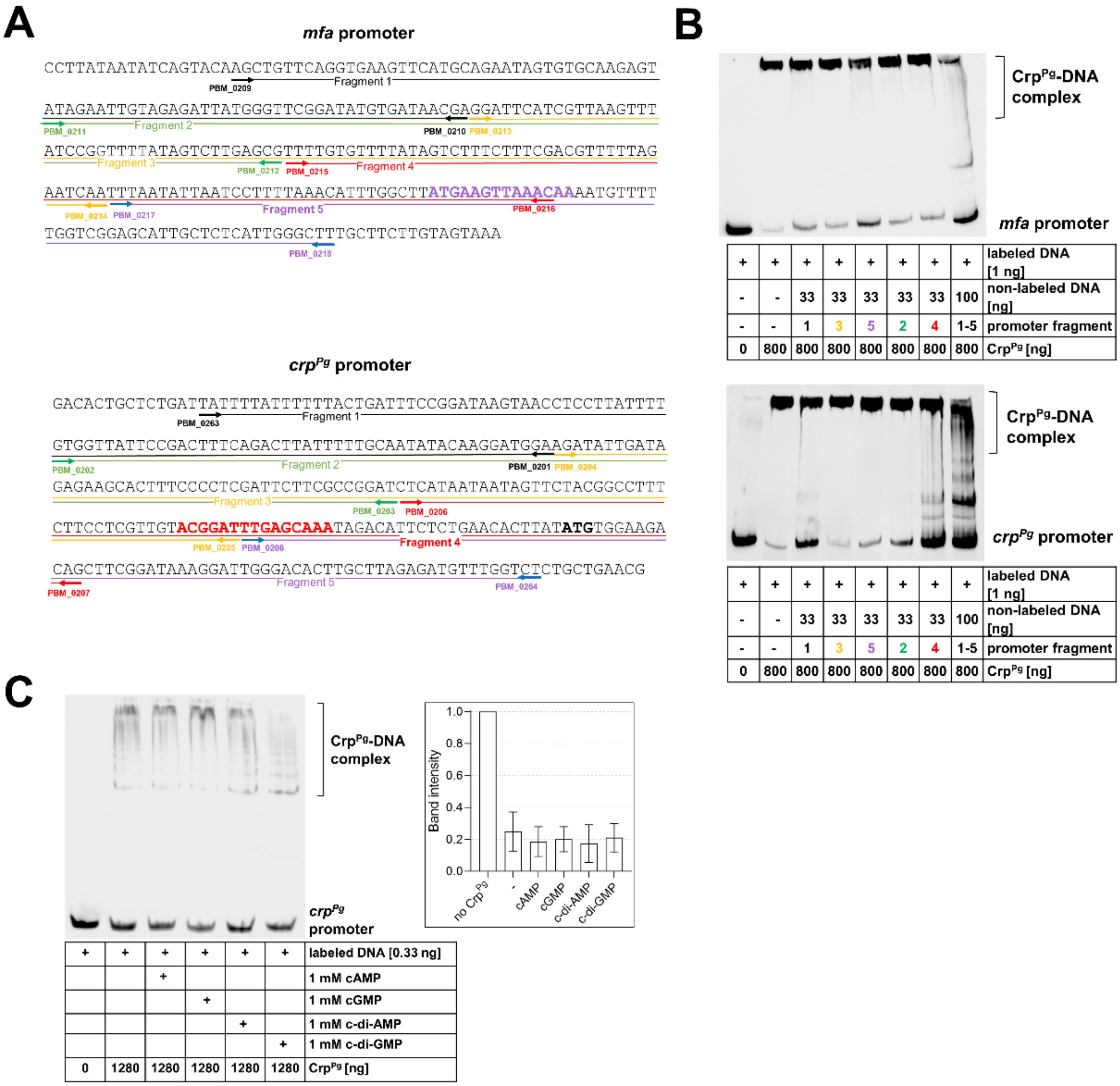

Fig. S4. Analysis of Crp^{Pg} binding to DNA. (A) Schematic presentation of promoter regions used as probes. Edges of the amplified promoter regions are marked with arrows and primer names listed in Table S2. The *crp^{Pg}* and *mfa* promoter fragments were analyzed. (B) EMSA was performed using biotin-labeled promoter region (comprising 1-5 fragments) obtained with primers PBM_0209 × PBM_0218 for *mfa* or PBM_0263 × PBM_0264 for *crp^{Pg}* and shorter unlabeled fragments (1, 2, 3, 4, or 5) in 100× excess for competition for Crp^{Pg} binding. The full unlabeled promoter region (comprising 1-5 fragments) was used as a control. The competitor promoter fragments that most significantly reduced the binding of Crp^{Pg} to the labeled promoter region were selected as those specifically bound by Crp^{Pg}. (C) Investigation of the influence of 1 mM cyclic nucleotides or di-nucleotides (cAMP, cGMP, c-di-AMP, and c-di-GMP) on Crp^{Pg}-DNA (promoter fragment 4) complex formation. The inset shows the relative band intensity of the free probe from 3 independent experiments.

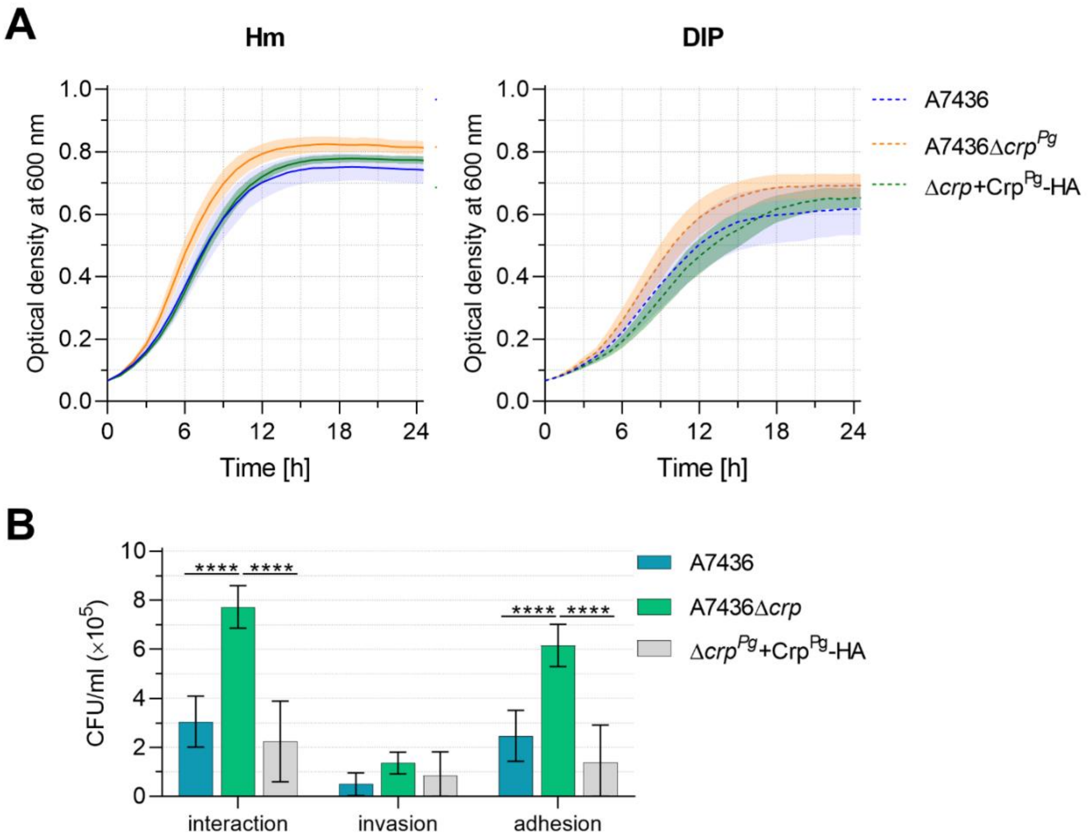

Fig. S5. Phenotypic characterization of the Δcrp^{Pg}+$\mathrm{Crp}^{\mathrm{Pg}}$-HA complemented strain. (A) The growth of bacteria in liquid culture media containing iron and heme (Hm) or without heme and supplemented with the iron chelator (2,2-dipyridyl; DIP) was monitored over time by measuring the optical density at 600 nm. (B) The ability to invade and adhere to host cells was analyzed using a *P. gingivalis*-gingival keratinocytes co-culture model. The number of viable bacteria was shown as the number of colony-forming units per 1 ml (CFU/ml). Adhesion – live bacteria attached to keratinocytes; invasion – live bacteria that invaded keratinocytes; interaction – the total number of live bacteria that invaded and adhered to keratinocytes. Experiments were performed three times in two biological replicates. Results are shown as mean±SE (A) or mean±SD (B). ****P<0.0001.

A

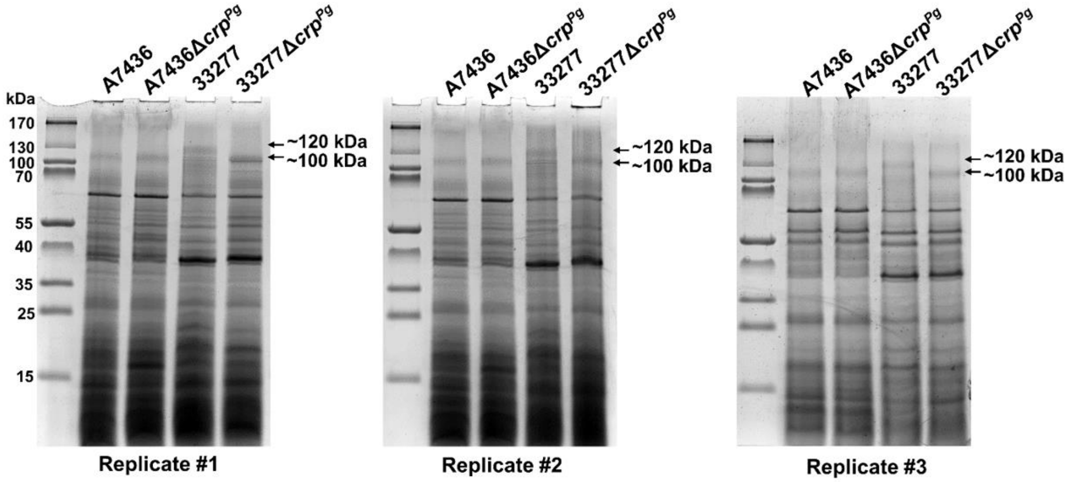

B

	Protein ID	Protein description	Protein MW (Da)	PLGS Score	No. of matched peptides	Protein sequence coverage (%)	No. of matched fragments	Protein relative amount (Top 3 peptides)
**~120 kDa**								
**33277** Replicate 1	P00761	Trypsin	24410	484	3	16	21	126691
	**F5HE64**	**Zinc carboxypeptidase putative**	**91518**	**239**	**10**	**7**	**40**	**147414**
**33277** Replicate 2	P00761	Trypsin	24410	467	3	16	30	496545
	**F5HE64**	**Zinc carboxypeptidase putative**	**91518**	**334**	**12**	**10**	**59**	**280310**
**33277Δ*crp*Pg** Replicate 1	P00761	Trypsin	24410	329	17	2	14	550800
**33277Δ*crp*Pg** Replicate 2	P00761	Trypsin	24410	177	4	21	22	14089
	**F5HE64**	**Zinc carboxypeptidase putative**	**91518**	**64**	**7**	**5**	**21**	**17743**
	Q7MWF4	Glycosyl hydrolase family 109 protein	52582	18	1	3	6	1283
**~100 kDa**								
**33277** Replicate 1	P00761	Trypsin	24410	389	2	8	21	1806171
	Q9RQJ2	Peptidylarginine deiminase	61730	117	8	10	28	40678
**33277** Replicate 2	P00761	Trypsin	24410	380	2	8	21	1189376
	**F5HE64**	**Zinc carboxypeptidase putative**	**91518**	**80**	**7**	**6**	**35**	**73129**
**33277Δ*crp*Pg** Replicate 1	P00761	Trypsin	24410	130	4	21	13	10004
	**F5HE64**	**Zinc carboxypeptidase putative**	**91518**	**46**	**9**	**8**	**28**	**14389**
**33277Δ*crp*Pg** Replicate 2	**F5HE64**	**Zinc carboxypeptidase putative**	**91518**	**1683**	**22**	**22**	**197**	**653700**
	P00761	Trypsin	24410	584	3	16	27	1543752

Fig. S6. Analysis of protein expression. (A) Protein production was examined in whole *P. gingivalis* wild-type and Δcrp^{Pg} mutant cell lysates by SDS-PAGE. After electrophoresis, proteins were visualized using CBB G-250. Bands corresponding to the mass of ~100 and ~120 kDa from the 33277 and 33277Δcrp^{Pg} strains (from replicates 1 and 2) were excised for MS analysis. (B) The table contains a summary of protein hits identified in each sample. The trypsin match (identified in each case) added externally to the sample was shown in grey.

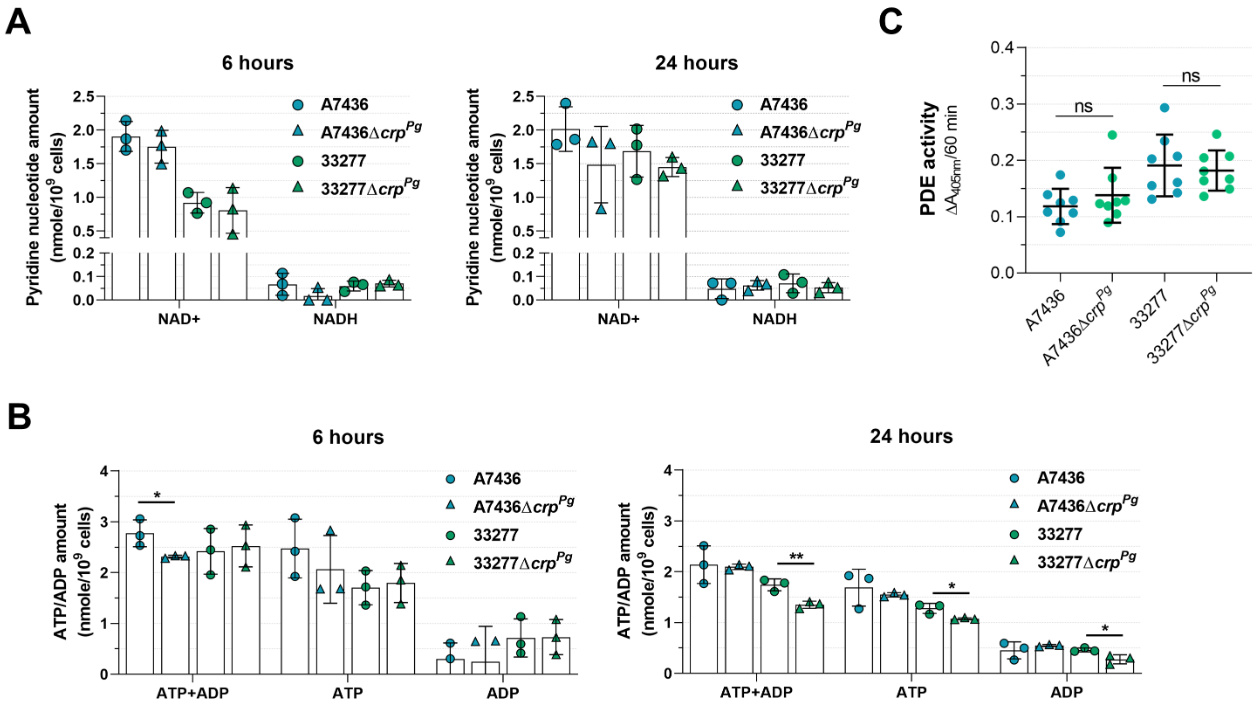

Fig. S7. Analysis of Crp^{Pg} influence on energy metabolism. Levels of NADH and NAD+ (A) and ATP and ADP (B) were determined in *P. gingivalis* cells after 6 and 24 h of growth in iron and heme-replete conditions (Hm medium). The nucleotide amounts were determined using the colorimetric-based NAD/NADH Assay Kit (Sigma-Aldrich) or the luminescence-based ADP/ATP Ratio Assay Kit (Sigma-Aldrich) according to the manufacturer's protocols which are based on the standard curves of the measured molecules. The results are shown as nmoles of respective molecules per 10^9 bacterial cells. (C) Determination of phosphodiesterase (PDE) activity was determined in *P. gingivalis* cells after 24 h of growth in the Hm medium using bis(*p*-nitrophenyl) phosphate sodium salt (Sigma-Aldrich) as a substrate. During the reaction, a product with an absorbance maximum at 405 nm (A_{405}) was released; therefore, the activity is shown as the increase in absorbance (ΔA_{405}) over 60 min. *$P<0.05$;**$P<0.01$; ns – statistically not significant.

Table S1. *Porphyromonas gingivalis* strains used in this study.

Strains	Description	Antibiotic resistance* (concentration)	References
A7436	wild-type strain	none	Laboratory collection
ATCC 33277 (33277)	wild-type strain	none	Laboratory collection
A7436Δ*crp*^{Pg}	A7436 strain with the deleted *crp*^{Pg} gene (PGA7_RS07240) - *crp*$^{Pg\Delta}$Em^{r}	erythromycin (3 µg/ml)	This study
33277Δ*crp*^{Pg}	ATCC 33277 strain with the deleted *crp*^{Pg} gene (PGN_1569) - *crp*$^{Pg\Delta}$Em^{r}	erythromycin (3 µg/ml)	This study
Δ*crp*^{Pg}+Crp^{Pg}-HA	A7436Δ*crp*^{Pg} mutant strain complemented with the pTIO-tetQ+crp^{Pg} plasmid encoding Crp^{Pg} protein with HA-tag at the C terminus	erythromycin (3 µg/ml), tetracycline (2 µg/ml)	This study

*Respective antibiotics were used for the selection of mutant strains. For other studies, Δ*crp*^{Pg}+Crp^{Pg}-HA complemented strain was grown in the presence of tetracycline (1 µg/ml) only.

Table S2. Primers designed and used in this study.

Primers	DNA sequence 5’→ 3’	Description	References
PBM_0531	GTAGTTATTGATGCCGGAGATAG	amplify upstream, flanking region of the *crp*^{Pg} gene (PGA7_RS07240, PGN_1569)	This study
PBM_0532	CGCATAACGGCTGGCCTCTAAGCAAGTGTCCCAATC		
PBM_0533	TGGGACACTTGCTTAGAGGCCAGCCGTTATGCGGCAG	amplify DNA fragment encoding erythromycin resistance cassette (*ermF*)	
PBM_0534	GATAATCTTGATCCTCTTGCCACCGCGGTTGTCTCTCTTTC		
PBM_0535	GAGAGACAACCGCGGTGGCAAGAGGATCAAGATTATCGAC	amplify downstream, flanking region of the *crp*^{Pg} gene (PGA7_RS07240, PGN_1569)	
PBM_0536	CACAGTTCCGCCATCAGC		
F-crp_ctr	CACTTCGAAGTTATTGTTCGCC	PCR confirmation of A7436Δ*crp*^{Pg} and 33277Δ*crp*^{Pg} mutant strains construction	This study
R-crp_ctr	TGCGGTGTACACAAGGACG		
F-ermF	GCCAGCCGTTATGCGGCAG		
PBM_0564	GATCCTCGAGAGAGTTTCCTTTCCGTGCCCAC	amplify *crp*^{Pg} (PGA7_RS07240) gene with the native promotor sequence; used to construct pTIO-tetQ+crp^{Pg} plasmid, allowing the production of Crp^{Pg} protein with the C-terminal HA fusion tag in the Δ*crp*^{Pg}+Crp-HA *P. gingivalis* complemented strain	This study
PBM_0271	GATCGGATCCTTAAGCGTAATCTGGAACATCGTATGGGTAACCGGAACGTGCGGTTTTTTGC		
PBM_0693	CTCGGGATCGAGGGAAGGATGTGGAAGACAGCTTCGGATAAAG	amplify *crp*^{Pg} gene (PGA7_RS07240), enabling cloning into pMAL_c5x_His plasmid	This study
PBM_0694	CCTGCAGGGAATTCGGATCCTTAACCGGAACGTGCGGTTTTTTG		
PBM_0741	CTTGACTTCAGTGGCGGCAG	amplify DNA fragment of the *P. gingivalis 16S rRNA* gene in RT-qPCR	Maeda et al., 2003
PBM_0742	AGGGAAGACGGTTTTCACCA		
PBM_0785	GAACGAAGAGGAACGAGAG	amplify DNA fragment of the *crp*^{Pg} gene (PGA7_RS07240) in RT-qPCR	Śmiga et al., 2019
PBM_0786	TAACGATGCGAGAAATGTG		
PBM_0761	TTCTGCGTTTGCCTTCTCCC	amplify DNA fragment of the *pgfur* gene (PGA7_RS06935) in RT-qPCR	Ciuraszkiewicz et al., 2014
PBM_0762	TGAGATCCTTGTCGGCCAGT		
PBM_0404	GATGGTTTCAACGAAAGCCC	amplify DNA fragment of the *mfa1* gene (PGA7_RS00800) in RT-qPCR	Śmiga et al., 2024
PBM_0405	GCTGCTGGTAGGAACGAAAC		
PBM_0398	CGGGTATCGTCCTGCTCATC		

PBM_0399	GAGCAGGGTTATGACGAGGG	amplify DNA fragment of the *PGA7_RS05995* gene in RT-qPCR	Śmiga et al., 2024
F_primer	ATGCAACGCATTGCCG	amplify DNA fragment of the *ustA* gene (PGA7_RS01095) in RT-qPCR	Boutrin et al., 2012
R_primer	TGCATTGGCTTCGCC		
PBM_0455	CTGCAGAAATTTTCTCGTTTTCC	amplify DNA fragment of the *hagC* gene (PGA7_RS08820) in RT-qPCR	This study
PBM_0456	TGATAGGCTCTGTCGCGTTC		
PBM_0980	CTGTAGAAAATTTGCGTCTGCAG	amplify DNA fragment of the *hagB* gene (PGA7_RS08805) in RT-qPCR	This study
PBM_0456	TGATAGGCTCTGTCGCGTTC		
PBM_0743	AGGTGTACTTGGCATTCCGTC	amplify DNA fragment of the *hagA* gene (PGA7_RS08180) in RT-qPCR	Ciuraszkiewicz et al., 2014
PBM_0744	CGTGTACGTGTAGTCGTTGGA		
PBM_0771	CTATCCTGGTGGGACTGCATC	amplify DNA fragment of the *fimA* gene (PGA7_RS09555) in RT-qPCR	Śmiga and Olczak, 2019
PBM_0772	ACCAAAGAATTGCCGAAAATC		
PBM_0263	TATTTTTTACTGATTTCCGGATAAGTAAC	amplify DNA fragments of the promoter of the *crp*^{Pg} gene used in EMSA; PBM_0263 and PBM_0206 primers were used with or without biotin attached to the 5' end of oligonucleotides	This study
PBM_0201	TCCATCCTTGTATATTGCAAAAATAAG		
PBM_0202	GTGGTTATTCCGACTTTCAGAC		
PBM_0203	ATCCGGCGAAGAATCGAG		
PBM_0204	AGATATTGATAGAGAAGCACTTTC		
PBM_0205	ATCCGTACAACGAGGAAG		
PBM_0206	CTCATAATAATAGTTCTACGGCC		
PBM_0207	CTGTCTTCCACATATAAGTGTTC		
PBM_0208	TTGAGCAAATAGACATTCTCTGAAC		
PBM_0264	AGACCAAACATCTCTAAGCAAGTG		
PBM_0209	AGCTGTTCAGGTGAAGTTCATG	amplify DNA fragments of the promoter of the *mfa1* gene used in EMSA; PBM_0209 and PBM_0217 primers were used with or without biotin attached to the 5' end of oligonuclotides	This study
PBM_0210	TCGTTATCACATATCCGAACCC		
PBM_0211	ATAGAATTGTAGAGATTATGGGTTCG		
PBM_0212	CGCTCAAGACTATAAAACCGG		
PBM_0213	GGATTCATCGTTAAGTTTATCCG		
PBM_0214	TGATTCTAAAAACGTCGAAAGAAAG		
PBM_0215	TTTTGTGTTTTATAGTCTTTCTTTCG		
PBM_0216	GTTTAACTTCATAAGCCAAATG		
PBM_0217	ATTTAATATTAATCCTTTTAAACATTTGGC		
PBM_0218	AGCCCAATGAGAGCAATGCTC		

Table S3. Gene expression in the Δ*crp*^{Pg} mutant strain constructed in the A7436 genetic background in comparison to the A7436 wild-type strain analyzed using transcript sequencing (RNA-seq). The experiment was carried out in bacteria grown under iron and heme-replete conditions (Hm medium) and collected in the mid-exponential growth phase (OD_{600} = 0.5-0.6). Genes with expression fold changes >1.5 (up-regulation) or <-1.5 (down-regulation) with adjusted *P* values (P_{adj}) <0.05 are listed.

Gene ID A7436	Gene name	Gene ID ATCC 33277	Gene ID W83	Gene description	Category	Fold change	P_{adj}-value
PGA7_RS09250		PGN_2053	PG2069	SDR family oxidoreductase	F	-3.26	<0.001
PGA7_RS10015	*rnpB*			RNase P RNA component class A	PS	-2.88	0.0015
PGA7_RS00165	*trxA*	PGN_0033	PG0034	thioredoxin	E	-2.54	<0.001
PGA7_RS09420		PGN_t0007	PG_t47	tRNA-Met	PS	-2.34	<0.001
PGA7_RS01095	*ustA*	PGN_0349	PG0246	up-regulated in stationary phase protein A, UstA	R	-2.29	<0.001
PGA7_RS03725		PGN_0927	PG1203	helix-turn-helix transcriptional regulator	R	-2.25	<0.001
PGA7_RS05335			PG1440	hypothetical protein	H	-2.19	<0.001
PGA7_RS07120		PGN_1544	PG0423	hypothetical protein/domain of unknown function (DUF4878)	H	-2.18	<0.001
PGA7_RS07825	*rpsO*	PGN_1698	PG1758	30S ribosomal protein S15	PS	-2.11	0.0051
PGA7_RS01470				hypothetical protein	H	-2.09	<0.001
PGA7_RS02310		PGN_0838	PG1491	hypothetical protein	H	-2.09	<0.001
PGA7_RS06615		PGN_1435	PG0536	hypothetical protein	H	-2.05	<0.001
PGA7_RS11865				2TM domain-containing protein	O	-2.04	<0.001
PGA7_RS09185		PGN_t0047	PG_t44	tRNA-Lys	PS	-2.03	<0.001
PGA7_RS06645		PGN_t0039	PG_t11	tRNA-Gly	PS	-2.02	<0.001
PGA7_RS00670	*secG*	PGN_0258	PG0145	pre-protein translocase subunit SecG	PF	-2.02	<0.001
PGA7_RS03980		PGN_1233	PG1135	sugar transferase	K	-2.02	<0.001
PGA7_RS08150		PGN_1739	PG1828	hypothetical protein	H	-2.00	0.0227
PGA7_RS11040				hypothetical protein	H	-1.97	<0.001
PGA7_RS05385			PG1451	nuclear transport factor 2 family protein	O	-1.93	<0.001
PGA7_RS08145		PGN_1740	PG1827	RNA polymerase sigma factor	R	-1.93	<0.001
PGA7_RS11240				hypothetical protein	H	-1.88	<0.001
PGA7_RS01705		PGN_0485	PG1634	hypothetical protein	H	-1.87	<0.001
PGA7_RS01310	*rpsT*	PGN_0394	PG1723	30S ribosomal protein S20	PS	-1.87	<0.001
PGA7_RS00790		PGN_0284	PG0173	winged helix DNA-binding protein	R	-1.84	<0.001
PGA7_RS10240		PGN_0895	PG1421	4Fe-4S binding protein	C	-1.82	<0.001

PGA7_RS00980				hypothetical protein	H	-1.79	0.0117
PGA7_RS05020		PGN_1040	PG0901	DUF4492 domain-containing protein	H	-1.79	<0.001
PGA7_RS06485	*efp*	PGN_0616	PG0568	elongation factor P	PS	-1.79	0.0134
PGA7_RS04195		PGN_1182	PG1085	hypothetical protein	H	-1.79	<0.001
PGA7_RS11495			PG1863	hypothetical protein	H	-1.79	0.0066
PGA7_RS07205		PGN_1561	PG0404	hypothetical protein	H	-1.78	<0.001
PGA7_RS00560		PGN_0235	PG0121	HU family DNA-binding protein	D	-1.77	<0.001
PGA7_RS11320		PGN_0273	PG0161	hypothetical protein	H	-1.77	<0.001
PGA7_RS07480	*megL*	PGN_1618	PG0343	methionine gamma-lyase	E	-1.76	<0.001
PGA7_RS10820	*ssrA*			transfer-messenger	PS	-1.74	<0.001
PGA7_RS02225			PG1513	histidine phosphatase family protein	B	-1.73	<0.001
PGA7_RS06585			PG0543	helix-turn-helix domain-containing protein	R	-1.73	0.0067
PGA7_RS05870		PGN_0746	PG0712	PAS domain-containing protein	R	-1.73	<0.001
PGA7_RS08085		PGN_1752	PG1813	4Fe-4S binding protein	C	-1.73	<0.001
PGA7_RS09545		PGN_0178	PG2130	DUF3575 domain-containing protein	H	-1.72	<0.001
PGA7_RS02550		PGN_0891	PG1416	nitronate monooxygenase	F	-1.71	<0.001
PGA7_RS11255				DUF1661 domain-containing protein	H	-1.69	0.0147
PGA7_RS09920		PGN_t0051	PG_t51	tRNA-Leu	PS	-1.68	<0.001
PGA7_RS05900		PGN_0740	PG0706	META domain-containing protein	C	-1.68	<0.001
PGA7_RS07705		PGN_1672	PG0292	chromate transporter	T	-1.68	<0.001
PGA7_RS04330		PGN_1298	PG1056	6-carboxytetrahydropterin synthase	B	-1.66	<0.001
PGA7_RS08375		PGN_1817	PG1890	hypothetical protein	H	-1.65	0.0026
PGA7_RS06700		PGN_1451	PG0521	co-chaperone GroES	PF	-1.64	0.0013
PGA7_RS06905		PGN_1496		DUF4248 domain-containing protein	H	-1.63	<0.001
PGA7_RS07580		PGN_1643	PG0318	DUF1573 domain-containing protein	H	-1.63	<0.001
PGA7_RS10120			PG1722	DUF1661 domain-containing protein	H	-1.62	<0.001
PGA7_RS07610		PGN_1650	PG0312	DUF4199 domain-containing protein	H	-1.61	<0.001
PGA7_RS07660		PGN_1660	PG0302	SoxR reducing system RseC family protein/positive regulator of sigma(E)	R	-1.61	<0.001
PGA7_RS07700		PGN_1671	PG0292	chromate transporter	T	-1.59	<0.001
PGA7_RS07710	*gldN*	PGN_1673	PG0291	gliding motility protein GldN	T	-1.58	<0.001
PGA7_RS00550		PGN_0233	PG0119	WecB/TagA/CpsF family glycosyltransferase	K	-1.58	0.0031
PGA7_RS02500	*xseB*	PGN_0840	PG1433	exodeoxyribonuclease VII small subunit	D	-1.58	<0.001
PGA7_RS07715	*gldM*	PGN_1674	PG0290	gliding motility protein GldM	T	-1.57	<0.001
PGA7_RS02210			PG1516	hypothetical protein	H	-1.57	0.0055

PGA7_RS02280			PG1501	TetR/AcrR family transcriptional regulator	R	-1.56	<0.001
PGA7_RS06775	*lipA*	PGN_1468	PG0504	lipoyl synthase	B	-1.55	<0.001
PGA7_RS03095		PGN_0092		helix-turn-helix domain-containing protein	R	-1.55	0.0488
PGA7_RS08250		PGN_1789	PG1856	dCMP deaminase family protein/cytidine and deoxycytidylate deaminase zinc-binding region	N	-1.54	<0.001
PGA7_RS06540				hypothetical protein	H	-1.54	0.0028
PGA7_RS03220		PGN_0068		hypothetical protein	H	-1.54	<0.001
PGA7_RS11715		PGN_1230	PG1133	hypothetical protein	H	-1.54	<0.001
PGA7_RS03100		PGN_0091		helix-turn-helix domain-containing protein	R	-1.53	0.0261
PGA7_RS06850	*yajC*	PGN_1485	PG0485	preprotein translocase subunit YajC	PF	-1.53	<0.001
PGA7_RS02195		PGN_0583	PG1519	DUF4209 domain-containing protein	H	-1.53	0.0049
PGA7_RS00180	*rplS*	PGN_0035	PG0037	50S ribosomal protein L19	PS	-1.53	<0.001
PGA7_RS04400		PGN_1313	PG1039	phosphoethanolamine transferase/sulfatase	O	-1.52	<0.001
PGA7_RS07140		PGN_1548	PG0419	DUF2807 domain-containing protein/putative auto-transporter adhesin, head GIN domain	H	-1.52	<0.001
PGA7_RS05380			PG1450	helix-turn-helix transcriptional regulator	R	-1.51	0.0143
PGA7_RS03290				hypothetical protein	H	-1.51	0.0032
PGA7_RS02475		PGN_0844	PG1458	DUF1896 domain-containing protein	H	-1.51	0.0085
PGA7_RS03125		PGN_0084		type IA DNA topoisomerase	D	1.50	<0.001
PGA7_RS08610	*rplP*	PGN_1861	PG1931	50S ribosomal protein L16	PS	1.51	<0.001
PGA7_RS09675		PGN_0203	PG2158	SufE family protein/Fe-S metabolism-associated domain	C	1.51	<0.001
PGA7_RS08510	*rpoA*	PGN_1840	PG1910	DNA-directed RNA polymerase subunit alpha	D	1.51	<0.001
PGA7_RS08620	*rplV*	PGN_1863	PG1933	50S ribosomal protein L22	PS	1.52	<0.001
PGA7_RS08615	*rpsC*	PGN_1862	PG1932	30S ribosomal protein S3	PS	1.52	<0.001
PGA7_RS07545		PGN_1636	PG0326	OprO/OprP family phosphate-selective porin	T	1.53	<0.001
PGA7_RS00075		PGN_0012	PG0016	sigma-54 dependent transcriptional regulator	D	1.53	<0.001
PGA7_RS02955		PGN_1112	PG1322	PhoH family protein	E	1.56	<0.001
PGA7_RS08095	*kdsB*	PGN_1750	PG1815	3-deoxy-manno-octulosonate cytidylyltransferase	K	1.56	<0.001
PGA7_RS03510		PGN_1389	PG1254	GNAT family N-acetyltransferase	C	1.56	<0.001
PGA7_RS11845			PG0382	hypothetical protein	H	1.57	<0.001
PGA7_RS08635	*rplW*	PGN_1866	PG1936	50S ribosomal protein L23	PS	1.58	<0.001
PGA7_RS08045	*atpB*	PGN_1761	PG1804	V-type ATP synthase subunit B	E	1.59	<0.001

PGA7_RS09060		PGN_1976	PG2029	zinc-dependent metalloprotease/domain of unknown function (DUF4953)	PF	1.60	<0.001
PGA7_RS02370	*traJ*		PG1479	conjugative transposon protein TraJ	O	1.60	0.0430
PGA7_RS02180	*menB*		PG1523	1,4-dihydroxy-2-naphthoyl-CoA synthase	B	1.62	0.0418
PGA7_RS08640	*rplD*	PGN_1867	PG1937	50S ribosomal protein L4	PS	1.62	<0.001
PGA7_RS01760	*bioC*	PGN_0469	PG1619	malonyl-ACP O-methyltransferase BioC	B	1.62	0.0199
PGA7_RS08595	*rplN*	PGN_1858	PG1928	50S ribosomal protein L14	PS	1.63	<0.001
PGA7_RS04675		PGN_0976	PG0976	phosphoribosylaminoimidazolesuccinocarboxa mide synthase	N	1.64	<0.001
PGA7_RS05985	*ftsE*	PGN_0721	PG0685	ABC transporter ATP-binding protein/ABC transporter	C	1.64	0.0057
PGA7_RS02360			PG1481	TraG family conjugative transposon ATPase	O	1.66	0.0243
PGA7_RS08655	*fusA*	PGN_1870	PG1940	elongation factor G	PS	1.66	<0.001
PGA7_RS09040	*rgpA*	PGN_1970	PG2024	Arg-gingipain RgpA/peptidase family C25	PF	1.66	<0.001
PGA7_RS08585	*rplE*	PGN_1856	PG1926	50S ribosomal protein L5	PS	1.66	<0.001
PGA7_RS02135			PG1533	bifunctional DNA primase/helicase	D	1.67	0.0364
PGA7_RS04900		PGN_1017	PG0930	hypothetical protein	H	1.67	0.0128
PGA7_RS07015	*thiF*	PGN_1516	PG0446	HesA/MoeB/ThiF family protein	E	1.67	<0.001
PGA7_RS00205	*nahA*	PGN_0039	PG0043	family 20 glycosylhydrolase/beta-hexosaminidase	K	1.70	<0.001
PGA7_RS07800		PGN_1692	PG1752	DUF3343 domain-containing protein	H	1.70	0.0026
PGA7_RS07925		PGN_1719	PG1779	O-acetyl-ADP-ribose deacetylase	D	1.71	<0.001
PGA7_RS08660	*rpsG*	PGN_1871	PG1941	30S ribosomal protein S7	PS	1.73	<0.001
PGA7_RS02175	*menD*		PG1524	2-succinyl-5-enolpyruvyl-6-hydroxy-3-cyclohexene-1-carboxylic-acid synthase	B	1.74	<0.001
PGA7_RS00730		PGN_0270	PG0158	ComF family protein/phosphoribosyl transferase domain	C	1.80	0.0015
PGA7_RS04670	*ubiE*	PGN_0975	PG0977	bifunctional demethylmenaquinone methyltransferase/2-methoxy-6-polyprenyl-1,4-benzoquinol methylase UbiE	B	1.81	<0.001
PGA7_RS00810	*mfa3*	PGN_0289	PG0180	fimbrial tip subunit Mfa3	K	1.83	<0.001
PGA7_RS08055	*atpI*	PGN_1759	PG1806	ATPase	E	1.85	<0.001
PGA7_RS06000		PGN_0718	PG0682	ABC transporter permease/FtsX-like permease family protein	T	1.85	0.0027
PGA7_RS02185			PG1522	o-succinylbenzoate synthase	B	1.90	0.0435

PGA7_RS07235		PGN_1568	PG0397	DUF721 domain-containing protein	H	1.95	<0.001
PGA7_RS11925				hypothetical protein	H	1.98	0.0199
PGA7_RS08050	*atpD*	PGN_1760	PG1805	V-type ATP synthase subunit D	E	1.99	<0.001
PGA7_RS00805	*mfa2*	PGN_0288	PG0179	fimbrial anchor subunit Mfa2	K	1.99	<0.001
PGA7_RS02285			PG1500	DUF2958 domain-containing protein	H	2.04	<0.001
PGA7_RS00800	*mfa1*	PGN_0287	PG0178	fimbrial major subunit Mfa1	K	2.06	<0.001
PGA7_RS02290	*topB*		PG1495	type IA DNA topoisomerase	D	2.14	0.0052
PGA7_RS08955		PGN_1953	PG2008	TonB-dependent receptor	T	2.19	<0.001
PGA7_RS05990	*ftsX*	PGN_0720	PG0684	ABC transporter permease/cell division FtsX-related transmembrane transport protein	C	2.20	<0.001
PGA7_RS06010		PGN_0716	PG0680	efflux RND transporter periplasmic adaptor subunit/HlyD family secretion protein	T	2.25	0.0456
PGA7_RS05150			PG0871	DUF3987 domain-containing protein	H	2.33	0.0116
PGA7_RS05995	*ftsX*	PGN_0719	PG0683	ABC transporter permease/cell division FtsX-related transmembrane transport protein	C	2.43	<0.001
PGA7_RS11885				hypothetical protein	H	3.29	0.0323
PGA7_RS11790				hypothetical protein	H	4.35	0.0143
PGA7_RS02140			PG1532	hypothetical protein	H	4.41	<0.001
PGA7_RS04970			PG0915	hypothetical protein	H	5.19	0.0434
PGA7_RS11785		PGN_0763	PG0732	hypothetical protein	H	5.78	<0.001
PGA7_RS11585				DUF1661 domain-containing protein	H	7.49	0.0119
PGA7_RS08820	*hagC*	PGN_1906	PG1975	hemagglutinin protein HagC	K	8.55	0.0024
PGA7_RS11175				DUF1661 domain-containing protein	H	14.02	0.0100

B – biosynthesis of cofactors/prosthetic groups; C – cellular processes; D – DNA/RNA processing; E – energy metabolism; F – fatty acid and phospholipid metabolism; H – hypothetical proteins; K – cell envelope; N – purines, pyrimidines, nucleosides, and nucleotides; O – other; PF – protein fate; PS – protein synthesis; R – signal transduction/regulatory functions; T – transport and binding proteins.

Table S4. Partial promoter sequences (unmarked regions) with 5' regions of open reading frames (shown in green). Potential Crp^{Pg}-binding boxes (sequence fragments shown in bold) were selected by comparing the promoter sequence with the DNA fragment recognized by Crp^{Ec} and those determined in *mfa*^{Pg} and *crp*^{Pg} gene promoters.

Gene name, locus ID in A7436 and W83 strains	DNA sequence
mfa1,<br>*PGA7_RS00800*,<br>*PG0178*,	CCTTATAATATCAGTACAAGCTGTTCAGGTGAAGTTCATGCAGAATAGTGTGCAAGAGTATAGAATTGTAGAGATTATG<br>GGTTCGGATATGTGATAACGAGGATTCATCGTTAAGTTTATCCGGTTTTATAGTCTTGAGCGTTTTGTGTTTTATAGTC<br>TTTCTTTCGACGTTTTTAGAATCAATTTAATATTAATCCTTTTAAACATTTGGCTT**ATGAAGTTAAACAAA**ATGTTTTT<br>GGTCGGAGCATTGCTCTCATTGGGCTTTGCTTCTTGTAGTAAAGAGGGCAATGGGCCCGATCCGGACAATGCGGCGAAG<br>TCGTATATGTCTATGACATTGTCCATGCCTATGGGAAGTGCTCGTGCGGGTGACGGACAGGATCAAGCTAACCCTGACT<br>ACCATTATGTAGGAGAGTGGGCAGGAAAAGACAAAATTGAGAAAGTGAGCATCTACATGG...
crp^{Pg},<br>*PGA7_RS07240*,<br>*PG0396*	GACACTGCTCTGATTATTTTATTTTTTACTGATTTCCGGATAAGTAACCTCCTTATTTTGTGGTTATTCCGACTTTCAG<br>ACTTATTTTTGCAATATACAAGGATGGAAGATATTGATAGAGAAGCACTTTCCCCTCGATTCTTCGCCGGATCTCATAA<br>TAATAGTTCTACGGCCTTTCTTCCTCGTTGT**ACGGATTTGAGCAAA**TAGACATTCTCTGAACACTTATATGTGGAAGAC<br>AGCTTCGGATAAAGGATTGGGACACTTGCTTAGAGATGTTTGGTCTCTGCTGAACGAAGAGGAACGAGAGCTATTAGAC<br>AAGGAGATACAACCTTTTCCTTGTAAGAAAGCCTCCACCGTCTTCTCTGAAGGAGACATCCCCAACAATCTTTTTTATC<br>TGTACGAAGGAAAAATAAAGATCCTGAGGGAAGGCGTATATGGACGCTTCCACATTTCTC...
PGA7_RS11175	GATTCGAACCACCGAAGGCGTAAGCCAGCTGAGTTACAGTCAGCCCCATTTGGCCACTCTGGTAACTGCCCATTGTTCT<br>TTTGCGATTGCAAAGGTAGAAACAAA**ATCCAATTATACAA**CAGTTCGCTCGGCAATTTTTTCTGCCATCTCTTTACGAA<br>CCGGAAAAACATCATCCATGGGGCCAAAAGATGTGGTTCGAGAATTTTTTCATTTTGGTTCGGGAAGTAAAAAATTCTC<br>GCGCCAAAACGAGAAAAAACTCGTTCGACTTTTTCCAATTTTACGAGCCGCAATCGGAGAGACTCCGGTGCGTAAATTT<br>TCAACCGATGGTTTGTATCGATAGAACAAAATCAGAATCATTTGCCCATCTTTCGGTCTGATGGG
trxA,<br>*PGA7_RS00165*,<br>*PG0034*	CGAACTGGTGAATAGATTGAAGACTTATGAGATTAACTTTGCACTTGAATAGACCA**ATCATTATAAACAA**GAATAACAA<br>CCAACTAAAACGAACAAAATTATGGCACTGCAAATTACAGATGCAACATTTGACGGCTTGGTAGCTGAAGGCAAGCCGA<br>TGGTAGTGGATTTCTGGGCTACATGGTGCGGTCCTTGCCGCATGGTAGGTCCGATCATCGACGAGCTGGCTGCGGAATA<br>TGAAGGACGCGCTATCATAGGAAAAGTGGATGTGGATGCAAATACGGAATTGCCCATGAAGTATGGCGTGCGTAATATC<br>CCCACCATTTTGTTCATCAAGAACGGCGAAGTGGTAAAGAAGCTCGTGGGTGCTCAGTCCAAAGACGTATTCAAGAAAG<br>AATTGGATGCCTTGTTTTAATTA
ustA,<br>*PGA7_RS01095*,<br>*PG0246*	GATAAAGAGCGGATAGAAGCTCCTGAGTTTTGGTATTGTCAAATAATATGTCTAAGTTAGGGGCAGAATAACAAACTGA<br>TTGTTGAACCACAAAAAAAGACGAAATTATTCCCATGACTAAGACAATCACATTCA**ATGAACTGAGAAGAATCAA**AGAC<br>AGCTTGCCCGATGGCGCTATGCAACGCATTGCCGATGAGTTGGGGCTGCGAGTGGAAACCGTACGCAACTATTTCGGCG<br>GTGCCAATTTCAGAGAAGGTCGTAGCTGCGGTGTTCATATCGAAGCAGGACCGGAAGGAGGTATTGTTGAGTTGGATGA<br>CACAACTATCTTAGAGAGGGCTTTGTCTATGCTGGGCGAAGCCAATGCATAGTCCTTTG

PGA7_RS03725, PG1203	GAATCCATAAAACAACGGAAATAAAGTTTCTTTCCTGCACCAATTAAGACGATTTAAGGGAATGGAAAATAAACCTCAT<br>TCCCTTTTTCTATGCTATTTCATTTCATATCTTTGCGCTATCAAAAGAACATTGAACGCGATGGATAAACCC**ATGAATC**<br>**GAATCAA**AGAAGTGCTTGAGGAGAAAGGCATCAAGCAAACCTGGCTTGCCGAAAAACTCGGCAAAAGCTTCAGCATTGT<br>CAATTCCTACGTCTGCAATCGCCGCCAACCAAGTTTGGACATATTGTTTGAAATTGCGAAGATATTAGAGGTGGACGTG<br>AGAGTGTTGATTGGCACTAAGGAAAACAGACAGAAGAGCAAATAAATTCAGAAGTT
PGA7_RS08955, PG2008	TTATCGTGGGATAGGGAGTGGGACACACTCTCCTAACCTCAAAAACCGACTAAAAAGGATCGGAATAAGGATACCGAAC<br>AGACACTATATCCATATCAAGCCAATCAAACCAAAAA**ATAAAATGAAACAA**CTAAACATTATCAGCTTCATCATTGCTT<br>TCCTATTCTTAGGAACGAGCGCATCGGCTCAGCAATCGGGCGGATCCGTTACAGGTACCGTAGTGGACAAAAGCTCAAA<br>AGAACCTATCGCATACGTACAAGTATTCGTCAAAGGAACCACTCTCGGAACTTCCACGGATGCAAACGGAAACTACTCG<br>ATCAAGGGAATCCCTTCGGGTAATCAAACTATCGTAGCCCGACTCATGGGTTACTCCACTTGCGAAGAAAAAGTACATA<br>TAGAAAAGGGTGGTTCCCGCCACGTAGACCTCTATCTGACCGAAGAGATTCTCTCTCTCGATGGGGTAGTGGTATCTGC<br>CAATAGAAACGAGACTTTCCGCCGTCAAGCACCCTCGTTGGTAACGGTACTGTCGCCGGAACTTTTCCTCAAAACCAAC<br>TCTACCAACCTGAGTC…
PGA7_RS06000, PG0682	AGCTCTGTGTACAAAAGAAACGATATGACTCTTATCCTTTTTCCTCTCTTGAAGAATCGAATCATGCGGATCTGTCTTA<br>TAAATTCATCCTTACAGAGTTGTAAAG**AGGAATTTATACAA**CGAAATTCCTCACTGCAAGCCTGCGATTATAAATTTCG<br>GAAGCGAAATTCCTCTCTGCAAGCTTGCAGGAAGCGATTTCGGCGAAAAAATTCCTATTTGCAGCTTGCAGGA**AGCGAT**<br>**TTCGGCAAA**AAAATTCCTATTTGCAGCTTGCAGGA**AGCGATTTCGGCAAA**AAAATTCCTGTTTGCAGCTTGCAAGAAGC<br>AATTTCGGCAAGAAAATTCCTGTTTGCAGGCCTGCGGCTATAAATCTCGGAAGCAAAATTCCTCTCTGCAAGCTTGCAG<br>CATGAAATTTCGACAAACGAAATTCCACACTGCAAGCCTGCAATATGAAATTTCGGCGAAAAAATTACTCGCTGCAAGC<br>TTGCGGCCATTAATCTCGGAAGCAAAATTTGTCACTGCAAGCATGTAACAGTATTTTCTGCAACCGAAATCACCGTCTG<br>CAAACTTGAATCAAAGAGATACTAATCATAAGACAATTATTTTCTCAATAAAATAATCATTACTTCCATATTTAGTCAT<br>AGCTATCATGCGTACTTATTTCAAATTCCTCAGCCGCAACAAACTCTACACCTTCGTTACCATCGTAGGCTTTGCCCTC<br>TCGCTCATATTCGTCTTGCTGCTTAGCTTCTATGTACGGCGCGAACAGCAAGCCGATCGTATTCATACCGATTACAAAC<br>GTATCTATCAGTACAATGTCGAGAGTAAGAATAACTGGAGCGGATTTATCTGTTGCTATCCTGCCGGAACGCTCATTCG<br>CGAGCAGGTACCCGATGTGGAAGAGATATGCCGCATCAGCGAATACAACGACAAGGAATACATCTTTATCGGTGCCGAC<br>AAGCAAAACGGGTTGATTGCCTCCCATCTTTCGGTCGAATCCAACTTCTTTACTTTCTTCGACGGCTATAAACTCCTTG<br>AGGGTGATCCCAAAACTGTCCTTTCCGAGCAGAACAGTGCCGTTATTTCCTCCGCGTTGGCAGCCCGTATCTTCGGTAA<br>CGTGTCTCCCATCGGGCAGGAGATTTCGTTTTTCGATTTTTCCAAAAACAAGCAGACCTTTCGGATCACGGGCATCATG<br>GAGCCGATGCCGGATAACTGCCATATCAGACCGGCCGAACTCCTTTTCTATCAGGAGCCCAAAGAAAA…
hagC, PGA7_RS08820, PG1975	ATTTTTTCATCTTACTATATTTTGGGATTATATTTCTACACCTCCTTATCCGGAATTTGGAAATGCGGGGCAAAAGTAG<br>AAAAATTTTATTTCCATCAAAAAAAATCTTCAATTTTTTTTCACTTTGCGCATTCTGCATATAAATGCTGCTACGTCGG<br>CAGATTATTCTGGTTAAAAAGTTATAGATGCAGCTCTTGGTTATAGTGTCCTAAGATCGCTATGCAACCTGTAAGAAAC<br>GATTGTAGGCTGTTTCTTGCTTCCTGCACGAATGCAGGAGAGCAGAAACGCCCGTTGCTGCTCCCGTCAATACACTAAT<br>TATTATCGACTTAACCCCTTAATTCAAAAACTAAAATGACTGCAGAAATTTTCTCGTTTTCCCGGCTCCAAAATTTGGA<br>GCACT**ACCGTTTTGCCAAGAA**TGTGCTGACGCTCTGTCGCACGGCAAATATCGCTAAACTGAATCCCAAACTGCCCGAG<br>CTGGAAAAGGCTATCGAAATGGAGGATTTGGCTCTGAATCCGCCCGTCGCGAACGAGCTGACGCCTCAGGTCATAGCCC<br>TCGACGAGGAACGCGACAGAGCCTATCAGGCGCTGATGTCGCGCGTGCGTTCGTATGCTTTCGACGAGGACAGCCAGCT

	GCGCAACGCGGCAGCCAGAATCGAAGACGTGGCCGCTCGCTACGGCAACGTGATCCGA**ATGAACTATGACAA**GGAGACGGCCGCGATAGAGAATTTCCTCACCGATCTCAAGGGCGAGAACATTCGCCCCCTCGTAACGAAACTCGGCGTGACGGCACTCGTTGACAGACTGGAAAAGAACAATAAGGCCTTCGCCGACTTCTTCCTCCGCCGTCTGAGCACCGACCAACGAGGCAAATATGACGTGAAGGCACTCCGTGCCGAGACCGACCGCACATTGGTAGCCGTGGTGCGCCGCATGGACTCCATCGACGACATGGAGCCGAGCCCGGAGATCCGTGCGCTCATCGAGCTCTACAACCGACTCGTGGCCAATCGCCGCGCCCTCTTGGC…
fur, PGA7_RS06935, PG0465	ATGGGCTATGGTACCTTGGGCGATCTTTATCCTACGGACAATTTCGGAATCTCGGAGCGAGGTATTACTTTCCTCTACAATGAGTACGAGATCGCTCCCTATGCCATGGGGCCGATACAGATTACGCTGTCGTTCGATTCGCTTGCTCCGATTCTCAAGGAGGATTCGCCCACTGCCTTGCTCTCTGCCGGCAAGAATGGATCGAAAAGATAAAAACCAATCGGGTTTCCTCGCGACCAACTTCTTCAAAAGAACTATATTTGTAGCTCCTGTCGATAGTGCCATGGCAGTTGTAAAGATGATAGTCACATCACTGGAAGATCTTCGCTCAAGATTGCGTGCATATGTGTCGGAGAACGGCTTGCGTCATACCCCCGAACGATACAGCATTCTTGAAGTCGCATATAATCTG**AAGAAGATATTCAC**GCCGGACGACTTGTTCGATCTCACTCGCGAGAATGGCTTGCCTGTAAGTCTTTCTACGGTCTATAATACCCTTACCTTGCTCGAACGCTGCGGGATCGTTCTGCGTTTGCCTTCTCCCGAAACCAAATATCAGTACTTGATGGCTTCATTTGCAGAGCAGTGTCCGCTGCTTTTCTGTACCGAATGTGCACAATTTTCTACCTACTACCGACGAAATGTGAAGTCGATACTGGCCGAC**AAGGATCTCAGACCA**CCACGCTTCTCTTATAGGCAGGCCATCATTTGCCTCTATGGTATATGCGACAAATGCAGGAAGAAGCAGTCGGCCCTCAAAAAGGCGGCGGACAAAGCCGCTCCCAAGAAGAAAAAATAATAACACAGAC

References

Boutrin, M.C., Wang, C., Aruni, W., Li., X. and Fletcher, H.M. (2012) Nitric oxide stress resistance in *Porphyromonas gingivalis* is mediated by a putative hydroxylamine reductase. *J. Bacteriol.*, 194, 1582-1592. doi: 10.1128/JB.06457-11.

Ciuraszkiewicz, J., Śmiga, M., Mackiewicz, P., Gmiterek, A., Bielecki, M., Olczak, M. and Olczak, T. (2014) Fur homolog regulates *Porphyromonas gingivalis* virulence under low-iron/heme conditions through a complex regulatory network. *Mol. Oral Microbiol.*, 29, 333-353. doi:10.1111/omi.12077.

Maeda, H., Fujimoto, C., Haruki, Y., Maeda, T., Kokeguchi, S., Petelin, M., Arai, H., Tanimoto, I., Nishimura, F. and Takashiba, S. (2003) Quantitative real-time PCR using TaqMan and SYBR Green for *Actinobacillus actinomycetemcomitans*, *Porphyromonas gingivalis*, *Prevotella intermedia*, *tetQ* gene and total bacteria. *FEMS Immunol. Med. Microbiol*., 39, 81-86. doi: 10.1016/S0928-8244(03)00224-4.

Śmiga, M. and Olczak, T. (2019) PgRsp is a novel redox-sensing transcription regulator essential for *Porphyromonas gingivalis* virulence. *Microorganisms*, 7(12), 623. doi:10.3390/microorganisms7120623.

Śmiga, M., Ślęzak, P., Tracz, M., Cierpisz, P., Wagner, M. and Olczak, T. (2024) Defining the role of Hmu and Hus systems in *Porphyromonas gingivalis* heme and iron homeostasis and virulence. *Sci. Rep.* (revised manuscript under review).

Śmiga, M., Stępień, P., Olczak, M. and Olczak, T. (2019) PgFur participates differentially in expression of virulence factors in more virulent A7436 and less virulent ATCC 33277 *Porphyromonas gingivalis* strains. *BMC Microbiol.*, 19(1), 127. doi:10.1186/s12866-019-1511-x.
